# Generation of unequal nuclear genotype proportions in *Rhizophagus irregularis* progeny causes allelic imbalance in gene transcription

**DOI:** 10.1111/nph.17530

**Published:** 2021-07-03

**Authors:** Chanz Robbins, Joaquim Cruz Corella, Consolée Aletti, Réjane Seiler, Ivan D. Mateus, Soon‐Jae Lee, Frédéric G. Masclaux, Ian R. Sanders

**Affiliations:** ^1^ Department of Ecology and Evolution University of Lausanne Biophore Building Lausanne 1015 Switzerland; ^2^ Group of Genetic Medicine Geneva University Hospital Building D Geneva 1205 Switzerland

**Keywords:** AMF genetics, arbuscular mycorrhiza, plant production, plant symbiosis, *Rhizophagus irregularis*

## Abstract

- Arbuscular mycorrhizal fungi (AMF) form mutualisms with most plant species. The model AMF *Rhizophagus* *irregularis* is common in many ecosystems and naturally forms homokaryons and dikaryons. Quantitative variation in allele frequencies in clonally dikaryon offspring suggests they disproportionately inherit two distinct nuclear genotypes from their parent. This is interesting, because such progeny strongly and differentially affect plant growth. Neither the frequency and magnitude of this occurrence nor its effect on gene transcription are known.
- Using reduced representation genome sequencing, transcriptomics, and quantitative analysis tools, we show that progeny of homokaryons and dikaryons are qualitatively genetically identical to the parent. However, dikaryon progeny differ quantitatively due to unequal inheritance of nuclear genotypes. Allele frequencies of actively transcribed biallelic genes resembled the frequencies of the two nuclear genotypes.
- More biallelic genes showed transcription of both alleles than monoallelic transcription, but biallelic transcription was less likely with greater allelic divergence. Monoallelic transcription levels of biallelic genes were reduced compared with biallelic gene transcription, a finding consistent with genomic conflict.
- Given that genetic variation in *R. irregularis* is associated with plant growth, our results establish quantitative genetic variation as a future consideration when selecting AMF lines to improve plant production.

## Introduction

Arbuscular mycorrhizal fungi (AMF; Glomeromycotina) are ubiquitous soil microorganisms that establish mutualistic relationships with most terrestrial plants (Van der Heijden *et al*., 1998; Davison *et al*., 2015; Brundrett & Tedersoo, 2018). Hyphae of these fungi absorb and transport soil inorganic nutrients, especially phosphorus and nitrogen, to plant roots (Govindarajulu *et al*., 2005; Fellbaum *et al*., 2012). In exchange, AMF receive photoassimilates and plant‐derived lipids (Bravo *et al*., 2017; Keymer *et al*., 2017). This symbiotic interaction occurs across the planet, making AMF global players in nutrient and carbon cycling, affecting plant growth and diversity (Van der Heijden *et al*., 1998; Bago *et al*., 2000; Steidinger *et al*., 2019). Accordingly, *Rhizophagus irregularis* is a model AMF species of Glomeracae, a dominant family of AMF communities (Tisserant *et al*., 2013; Rodriguez‐Echeverria *et al*., 2017; Gao *et al*., 2019).

AMF are coenocytic, with thousands of nuclei coexisting within a common cytoplasm. Recent studies show that *R. irregularis* isolates are either homokaryons or dikaryons and that nuclei are haploid (Ropars *et al*., 2016; Wyss *et al*., 2016; Chen *et al*., 2018b; Masclaux *et al*., 2018, 2019). Dikaryon AMF harbour populations of two genetically distinct nuclei; referred to hereafter as nuclear genotypes (Masclaux *et al*., 2018). Although evidence implies that *R. irregularis* might reproduce sexually, population genetic studies suggest that clonal reproduction occurs frequently in nature (Ropars *et al*., 2016; Savary *et al*., 2018a; Mateus *et al*., 2020). Indeed, *R. irregularis* isolates have been maintained clonally for almost 20 yr *in vitro* (Koch *et al*., 2004; Rosikiewicz *et al*., 2017). Since initiating cultures, dikaryon isolates continually retain both nuclei (Angelard *et al*., 2010; Ropars *et al*., 2016; Wyss *et al*., 2016; Masclaux *et al*., 2018, 2019)

**Fig. 1:**
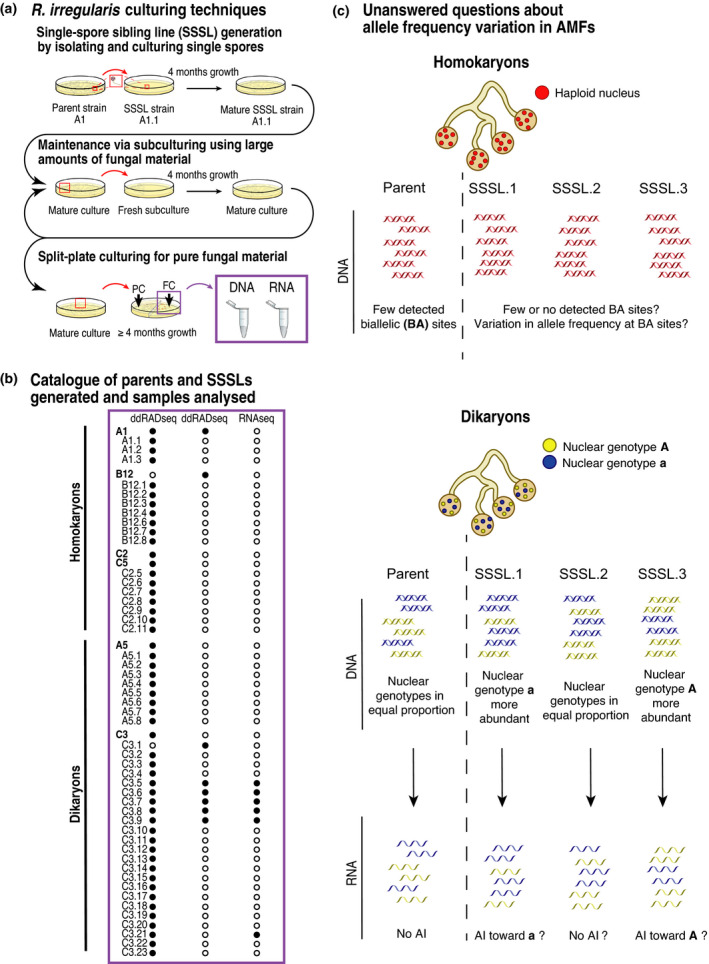
Experimental procedures, collected data, and unanswered questions about genetic variation by clonally produced arbuscular mycorrhizal fungus (AMF) siblings. (a) Single‐spore culturing of *Rhizophagus* *irregularis* involves taking one spore to initiate a new culture and produce sibling cultures of a parental isolate. Subculturing involves transferring a large amount of fungal material to maintain single‐spore sibling lines (SSSLs) and produce a larger amount of material for molecular analyses. PC, plant compartment; FC, fungal compartment. (b) Parental AMF lines and their SSSLs that were used for molecular analyses. Black dots indicate samples included in a given analysis, and white dots indicate the samples were not used for a given analysis. (c) Schematic diagram of the unanswered questions posed in this study about generation of genetic variation among SSSLs and their gene transcription. Analysis at the genome level using DNA sequencing allows the test of whether siblings of a parental AMF isolate are genetically indistinguishable (as expected in homokaryon offspring) or genetically variable at the quantitative level (as was predicted in dikaryon offspring), while transcriptome analysis allows the test of whether allelic imbalance (AI) in gene transcription occurs in dikaryon siblings that display quantitative genetic variation at the genome level.

Single‐spore culturing is a technique that allows the generation of AMF single‐spore sibling lines (SSSLs) from an AMF isolate that we call here a parental isolate (Fig. 1a). At present, unequal inheritance of nuclear genotypes has only been described for a small number of SSSLs from one parental isolate, known as C3 (Croll *et al*., 2009; Ehinger *et al*., 2012; Angelard *et al*., 2014; Masclaux *et al*., 2018). Three studies demonstrated that SSSLs of C3 (with an approximate 1 : 1 ratio of the two nuclei) can inherit different proportions of both nuclear genotypes (Angelard *et al*., 2010, 2014; Masclaux *et al*., 2018). The first study detected genetic variation among SSSLs by assessing amplified fragment length polymorphisms (Angelard *et al*., 2010). As discussed by Angelard *et al*. (2010), although this multilocus technique can verify the presence of an allele, it lacks sufficient sensitivity to measure changes in allele frequency or among SSSLs and does not distinguish single‐copy from multicopy regions of the genome. Both capillary electrophoresis and amplicon sequencing at a single locus (known as the *bg112* locus) confirmed that nuclear genotypes can be unequally inherited among SSSLs of C3 (Angelard *et al*., 2010; Ehinger *et al*., 2012; Masclaux *et al*., 2018). These results indicated that significant shifts in allele frequencies sometimes arise among clonally produced dikaryon spores. The study by Masclaux *et al*. (2018) considered one single‐copy locus in few SSSLs, where PCR error represents an unlikely, but possible, source of variation. A high‐throughput, reduced‐representation approach, such as double‐digest restriction‐site‐associated DNA sequencing (ddRADseq), is a reliable method for detecting genetic diversity within populations and distinguishing quantitative differences in allele frequencies (Wyss *et al*., 2016).

Whole‐genome amplification and sequencing of individual nuclei were fundamental in establishing that *R. irregularis* isolates have either a homokaryon or dikaryon genome organization (Lin *et al*., 2014; Ropars *et al*., 2016; Chen *et al*., 2018a). Yet, this technique offers too low resolution and, thus, is not well suited for determining quantitative differences in allele frequencies among several lines. First, the success rate of obtaining data of sufficient quality from a single‐nucleus of *R. irregularis* is staggeringly low, ranging from 10 to 63% (Lin *et al*., 2014; Ropars *et al*., 2016; Chen *et al*., 2018a). Second, the quantity of data needed to address quantitative variation at multiple loci among several lines, is fiscally prohibitive. For example, to date, < 300 nuclei have been sequenced, of which, only 148 passed quality filters: four in Lin *et al*. (2014); 59 in Ropars *et al*. (2016); and 85 in Chen *et al*. (2018a). A conservative assessment of changes in nuclear dynamics among dikaryon SSSLs would require a minimum of 1000 successfully sequenced nuclei from each SSSL. For this reason, ddRADseq is more suitable to estimate quantitative genetic variation existing among SSSLs at multiple biallelic sites across the genome. By using ddRADseq, allele frequency variation can be estimated in many dikaryon and homokaryon SSSLs and compared with their parent to quantify changes in nuclear dynamics. The premise of this analysis rests on the fact that biallelic sites must be single‐copy regions of the genome, meaning that detection of two alleles would only be possible if two different nuclear genotypes were represented. Thus, estimating the frequency of the two alleles can serve as a proxy for the relative abundance of both nuclear genotypes in a dikaryon. It is true that some biallelic sites were still detected in ddRADseq and whole‐genome sequencing of homokaryons (Wyss *et al*., 2016; Chen *et al*., 2018b; Savary *et al*., 2018b; Masclaux *et al*., 2019). However, these loci are very few, located in problematic regions of the assembly, and seem to have no discernible functional consequence (Masclaux *et al*., 2019). In stark contrast, biallelic sites in dikaryon C3 were more prevalent, and likely impact biallelic gene expression (Masclaux *et al*., 2019).

Quantitative genetic variation among SSSLs is likely significant for their symbiotic interaction with plants, since SSSLs differ significantly in fungal quantitative traits, how they colonize roots, and how they affect plant biomass (Ehinger *et al*., 2012; Angelard *et al*., 2014; Savary *et al*., 2018a). Indeed, pot experiments with rice, as well as field studies with cassava, indicate that genetic variation among SSSLs has enormous effects on plant biomass (Angelard *et al*., 2010; Ceballos *et al*., 2013, 2019; Mateus *et al*., 2019; Savary *et al*., 2020). The link between qualitative genetic variation (presence or absence of single‐nucleotide polymorphisms (SNPs)) of *R. irregularis* isolates and plant growth was recently presented by Ceballos *et al*. (2019), although likely depends additionally on plant host, edaphic characteristics, and other biotic and abiotic factors. However, the more elusive link between quantitative genetic variation (allele proportions) among SSSLs and its effect on plant growth has not yet been made. It is first necessary to understand whether quantitative changes in nuclear dynamics lead to quantitative differences in gene expression of dikaryon SSSLs.

How often, and by how much, quantitative differences in allele frequencies vary among dikaryon SSSLs could have profound consequences on fungal gene expression and on the AMF–plant symbiosis. For example, imbalanced nuclear ratios affect gene transcription and growth rate of the heterokaryon basidiomycete *Heterobasidion parviporum*, resulting in phenotypic differences from true diploid individuals (Clergeot *et al*., 2019). Moreover, owing to intricacies of transcriptional regulation within nuclei, equal proportions of two nuclei may not necessarily result in equal allele transcription. This may be due to localized transcriptional bursts, allele‐specific gene imprinting, or other mechanisms (Dong *et al*., 2017; Lafon‐Placette *et al*., 2018; Larsson *et al*., 2019). For example, the dikaryon basidiomycete *Agaricus bisporus* exhibits imbalanced allele expression at different growth stages, despite both nuclear genotypes being equally abundant (Gehrmann *et al*., 2018). Interestingly, many biallelic sites in the dikaryon *R. irregularis* isolate C3 were expressed in proportions equal to nuclear genotype proportions estimated from ddRADseq data, as well as the frequencies of both *bg112* alleles (Masclaux *et al*., 2018). Although this study showed that both nuclei were transcriptionally active in dikaryons, it could not address the effects of unbalanced nuclear dynamics on the contribution of gene expression in SSSLs from each of the two different nucleus genotypes. To address that, gene transcription needs to be assessed among SSSLs that have variable proportions to the two nuclear genotypes. Our current knowledge of how frequent nuclear genotype proportions vary in *R. irregularis*, as well as consequences on transcription, is very limited (Kokkoris *et al*., 2020, 2021; Yildirir *et al*., 2020).

To assess quantitative variation at biallelic sites, 48 SSSLs were generated from three homokaryon and two dikaryon ‘parental’ isolates of *R. irregularis* (Fig. 1a,b). The parental isolates represent single‐spore cultures from a field in Switzerland, and have been propagated clonally in axenic conditions for almost 20 yr (Koch *et al*., 2004). We employed ddRADseq to study allele frequencies at hundreds of biallelic sites to test the prevalence and amplitude of quantitative genetic variation among SSSLs (Fig. 1b,c). We later focused on six dikaryon SSSLs of C3 to investigate allelic imbalance in expressed genes at single‐copy biallelic sites and whether allele proportions reflect nuclear dynamics (Fig. 1c). Here, we define allelic imbalance to mean unequal transcription of two alleles of single‐copy biallelic genes, such that the two alleles are located on different nuclear genotypes and transcription of each allele represents the transcriptional contribution of each nuclear genotype. We hypothesized that transcribed alleles at biallelic sites would reflect DNA allele frequencies detected with ddRADseq. We investigated allelic expression patterns across hundreds of biallelic sites to further test whether all biallelic genes in an *R. irregularis* dikaryon displayed biallelic expression (i.e. both copies expressed) or whether some genes only exhibited monoallelic expression.

## Materials and Methods

### Fungal material and growth conditions

*Rhizophagus irregularis* isolates from Tänikon, Switzerland (‘parental’ isolates; homokaryons: A1, B12, C2; and dikaryons: A5 and C3), were used in this study (Koch *et al*., 2004). C5 was also included in the analysis and is considered a clone of C2 as they are genetically indistinguishable (Wyss *et al*., 2016; Savary *et al*., 2018a). Forty‐eight SSSLs were generated from these parents and maintained at 25°C in dark, axenic conditions with Ri T‐DNA‐modified carrot roots (Fig. 1a) (St‐Arnaud *et al*., 1996; Rosikiewicz *et al*., 2017). Additional cultures were produced independently for conducting a second ddRADseq (five SSSLs of C3), as well as RNA sequencing (RNAseq; six SSSLs of C3), and were maintained in the same manner (Fig. 1b). Three individual split plates (three biological replicates; ddRADseq) or three pools each of four split plates (three biological replicates; RNAseq) were produced.

### DNA extraction, double‐digest restriction‐site‐associated DNA sequencing library preparations, and sequencing

After at least 4 months, medium from fungal compartments containing AMF hyphae and spores was dissolved in 500 ml stirred citrate buffer (0.0062 M citric acid, 0.0028 M sodium citrate) for 20 min. One compartment represented one biological replicate (Fig. 1a). Fungal material was collected, flash frozen, and stored at −80°C until use.

Homogenized samples (CryoMill; Retsch GmbH, Haan, Germany) (2× 30 s, 25 Hz, resting 30 s, 5 Hz) were used to extract DNA (Qiagen Plant DNA kit; Qiagen, Hombrechtikon, Switzerland). DNA was quantified (Promega Quantus™ Fluorometer and DNA QuantiFluor^®^ dye; Promega AG, Dübendorf, Switzerland) and stored at −20°C.

During ddRADseq library preparation, samples were subjected to duplicate digests to obtain two technical replicates of each sample (2 h at 37°C, then 20 min at 65°C: 1× CutSmart^®^ buffer, 50 mM sodium chloride (NaCl), 0.05 µg µl^−1^ BSA, 1 U *Mse*I, 5 U *Eco*RI‐HF^®^, 6 µl template) using a frequent (*Mse*I: New England Biolabs, Bioconcept AG, Allschwil, Switzerland) and a less frequent (*Eco*RI‐HF^®^: New England Biolabs, Bioconcept AG) cutting restriction enzyme(Wyss *et al*., 2016; Savary *et al*., 2018a). DNA was diluted to 15 ng µl^−1^, or used directly at lower concentrations. Adapters and barcodes were ligated (6 h at 16°C, then 10 min at 65°C: 1× T4 ligase buffer, 14 mM NaCl, 0.014 µg µl^−1^ BSA, 862 nM *Mse*I adapter, 86.3 nM *Eco*RI adapter, and 335 U T4 ligase; Supporting Information Table S1) and samples were purified (AMPure XP beads; Beckman‐Coulter, Indianapolis, IN, USA; 1× bead volumes) before PCR. PCRs were performed in triplicate (30 s 98°C, 26 cycles (20 s 98°C, 30 s 60°C, 40 s 72°C), followed by 10 min 72°C; 1× Q5^®^ High Fidelity Buffer, 363 µM dNTPs, 305 nM forward and reverse primers (Table S1), 0.9× High GC Enhancer, and 0.4 U Q5^®^ High Fidelity polymerase), verified by gel electrophoresis (1.5% agarose gel, 100 V for 1 h), size selected (*c*. 300 bp; AMPure, 1× bead volume), and quantified before pooling. Equal quantities of ≤ 48 samples were pooled per library (Table S2). Libraries were purified (AMPure, 1× bead volume) and verified (Fragment Analyzer; Agilent, Santa Clara, CA, USA) before sequencing.

The five SSSLs of C3 underwent the same procedure, but with doubled reaction volumes and were pooled and sequenced independently in a single library (Fig 1b).

Lausanne Genomic Technologies Facility sequenced 100 bp paired‐end reads using Illumina^®^ HiSeq 2500 (Illumina, San Diego, CA, USA). Demultiplexed data files are deposited with European Nucleotide Archive under accession nos. PRJEB37069 (parental isolates and 48 SSSLs) and PRJEB39082 (five SSSLs of C3).

### RNA extraction, RNA‐sequencing library preparation, and sequencing

After 4 months, medium from fungal compartments of six SSSLs of C3 were dissolved in stirred citrate buffer for 50 min and washed with sterile double‐distilled water. Four pooled compartments represented one biological replicate. Total RNA was extracted (Maxwell RSC Plant RNA kit; Promega) and RNA quantity and quality were determined (Nanodrop photometer and Agilent 5200 Fragment Analyzer). Two duplicate RNAseq libraries were prepared using 100 ng RNA each and 13 cycles of PCR enrichment, representing technical replicates of each biological replicate (NEBNext Ultra II RNA Library Prep Kit for Illumina; New England Biolabs). Libraries with unique indices were pooled, and 150 bp paired‐end reads were sequenced with an Illumina HiSeq 4000 platform in three lanes. Six replicates (two technical replicates of three biological replicates) of each *R. irregularis* SSSL were sequenced. RNAseq reads were deposited in the European Nucleotide Archive (PRJEB39188).

### Double‐digest restriction‐site‐associated DNA sequencing data preprocessing on six parental isolates and 48 single‐spore sibling lines

Low‐quality reads were removed using Casava filter (Y). Adapters and low‐quality bases were trimmed using tagcleaner.pl (Schmieder *et al*., 2010). Only paired reads with lengths > 50 bp, and mean base quality > 25, were retained with prinseq‐lite‐0.20.4 (Schmieder & Edwards, 2011). Demultiplexing was done with process_radtags, allowing ≤ 2 bp mismatch (Catchen *et al*., 2011). Sample reads were mapped to *R. irregularis* DAOM197198 (ASM43914v3) to assess qualitative differences. Quantitative analyses of allele frequencies at biallelic sites were achieved by mapping to the respective parental genome (PRJEB33553). Only uniquely mapped reads were considered using the bwa mem algorithm with –c 2 (Table S2; Li & Durbin, 2009). Variants with allele frequency ≥ 10% and coverage ≥ 20 were called using Freebayes v.1.2.0, and only biallelic sites were considered further (Garrison & Marth, 2012). Variable sites were filtered (present in ≥ 60% of biological replicates) with BCFtools to obtain common variants (Li *et al*., 2009). All scripts are available at https://github.com/chanz06/AMF_RADseq_scripts.

### Qualitative and quantitative analyses of double‐digest restriction‐site‐associated DNA sequencing data on parental isolates and 48 single‐spore sibling lines

Samples containing ≥ 4000 SNPs were combined in a presence/absence matrix (165 303 sites; missing information was considered as absent). These filters eliminated the parent isolate B12 from further analyses. The dendextend v.1.14.0 and circulize v.0.4.10 R packages computed distances and generated a phylogenetic tree using the binary distance method. The package gmodels v.2.18.1 was used to compute principal components using the fast.prcomp function.

Common biallelic sites among a parent and all its SSSLs were selected, and reads supporting the reference and alternative allele were used to compute allele frequencies. Allele frequencies at biallelic sites were quantitatively assessed using two methods. First, a traditional chi‐squared test was used to detect significant differences between the reference allele abundance in the parent and SSSL at each biallelic site. Second, a nonparametric Mann–Whitney *U*‐test was used to test quantitative changes in allele frequencies between SSSLs and their parent. All biallelic sites and statistical testing results are documented in Table S3(a–d). Mean reference allele frequencies were finally tested with a one‐sample *t*‐test for significant shifts in SSSLs compared with their parent. All scripts are available at: https://github.com/chanz06/AMF_RADseq_scripts.

### Double‐digest restriction‐site‐associated DNA sequencing data preprocessing and mapping of five dikaryon single‐spore sibling lines of C3

Adapter sequences were removed with tagcleaner.pl and low‐quality reads were trimmed with prinseq.pl (Schmieder *et al*., 2010; Schmieder & Edwards, 2011). Only reads ≥ 50 bp were kept. Reads were aligned to *R. irregularis* A4 genome (PRJNA299206), using novoalign v.3.04.04 (Novocraft Technologies, Selangor, Malaysia). This assembly was used because previous analyses revealed high similarity to C3, and thus these two isolates are considered genetically indistinguishable (Wyss *et al*., 2016; Savary *et al*., 2018a; Chen *et al*., 2018b). Mapping statistics can be found in Table S4.

The same exact methods were applied to diploid (*Candida albicans* and *Betula* *nana*) and tetraploid (*Betula* × *intermedia*) controls. Publicly available ddRADseq data are retrievable from the National Center for Biotechnology Information Sequence Read Archive database (BioProject accession nos. PRJNA268659 and PRJEB3322) using the reference genomes GCA_000182965.3 and GCA_000327005.1 (Wang *et al*., 2013).

### RNA‐sequencing data preprocessing and mapping of six dikaryon single‐spore sibling lines of C3

Adapter sequences and low‐quality bases were removed with trimmomatic v.0.36 (Bolger *et al*., 2014). Reads were mapped onto the A4 genome (PRJNA299206) with Star software v.2.6.0, using the following parameters: ‐‐alignIntronMin 20 ‐‐alignIntronMax 5000 ‐‐outFilterMismatchNoverLmax 0.4 ‐‐outFilterMismatchNmax 15 ‐‐sjdbOverhang 99 ‐‐outFilterIntronMotifs RemoveNoncanonical ‐‐alignEndsType EndToEnd ‐‐outSAMtype BAM SortedByCoordinate ‐‐outSAMattributes Standard ‐‐outSAMstrandField intronMotif (Dobin *et al*., 2013). Mapping statistics are contained in Table S5.

### Variant calling, filtering, and allele frequency estimations of biallelic sites of single‐spore sibling lines of C3 from double‐digest restriction‐site‐associated DNA‐sequencing and RNA‐sequencing data

Variant calling was performed in the same way for both data sets using Freebayes v.1.2.0 (Garrison & Marth, 2012). SNPs, indels, and multiple‐nucleotide polymorphisms were detected with coverage > 10 with a diploid assumption (‐p 2). Parameters ‐0 ‐J ‐K ‐u ‐F 0.1 ensured all possible variants were called.

SNPs in repeats were discarded using a repeat annotation file and ‘bedtools intersect’ (Quinlan, 2014). Only biallelic sites with a Phred‐scaled Qscore ≥ 30 and in scaffolds > 1 kb were considered, provided they were detected in all six replicates (Tables S6, S7). Finally, only biallelic sites with a depth within the interquartile range of its sample, and ≥ 20 reads and in at least five replicates, were retained. We estimated the pooled reference allele frequency at all biallelic sites that did not vary significantly among replicates (chi‐squared test, *P* > 0.05). Statistics and plots were performed using R software (Ihaka & Gentleman, 1996). A principal component analysis (PCA) of common biallelic sites in ddRADseq data was performed with the prcomp function. Plots were made with ggplot2 package. Custom Python scripts are available at https://github.com/jquimcrz/afreq_NGS.

### Allelic fold‐changes and allelic imbalance thresholds of genes in RNA‐sequencing data

To investigate genes with evidence of disproportionate allelic transcription, log_2_ values of allelic fold‐change (aFC) were estimated for each gene containing at least one biallelic site (passing filters). When a gene contained > 1 biallelic position, the highest coverage position was used for estimates, meaning that aFC was based on allele frequencies at one biallelic site. Thresholds applied to determine allelic imbalance were absolute values of log_2_(aFC) > 0.5. A chi‐squared test compared the proportion of genes under allelic imbalance among SSSLs using prop.test. A *post hoc* pairwise comparison of proportions was performed with pairwise.prop.test, using Holm–Bonferroni corrections.

### Functional annotation of genes under allelic imbalance

We used eggNOG mapper to perform a functional annotation of identified genes under allelic‐imbalance (Jensen *et al*., 2008; Huerta‐Cepas *et al*., 2019). Results were summarized based on their clusters of orthologous groups (Tatusov *et al*., 2003).

### Identification of biallelic sites with monoallelic and biallelic expression

The genomic biallelic sites were first identified by mapping whole‐genome sequencing reads of A4 (PRJNA299206) to the A4 assembly (novoalign v.3.04.04) and calling variants using the same parameters as described for six SSSLs (Garrison & Marth, 2012). Only biallelic sites in coding sequences of annotated genes, with ≥ 25 depth and ≥ 10 reads supporting both alleles, were considered further.

Using ‘samtools depth’, we then computed the number of reads mapping to biallelic positions (≥ 25 reads) from each RNAseq alignment file and looked for the presence of either one (monoallelic expression) or two (biallelic expression) alleles among mapped reads in these biallelic sites (Li *et al*., 2009).

### Fragments per kilobase of transcript per million mapped reads analysis of genes with monoallelic and biallelic expression

Fragments per kilobase of transcript per million mapped reads (FPKM) values were computed for genes using Rseqc ‘FPKM_count.py’ (Wang *et al*., 2012). Significant differences (*P* < 0.05) between monoallelic and biallelic expressed genes were determined by Mann–Whitney *U*‐test of transformed values (log_2_(FPKM + 1)).

## Results

### Qualitative assessment of *Rhizophagus irregularis* parental isolates and their 48 single‐spore sibling lines

**Fig. 2:**
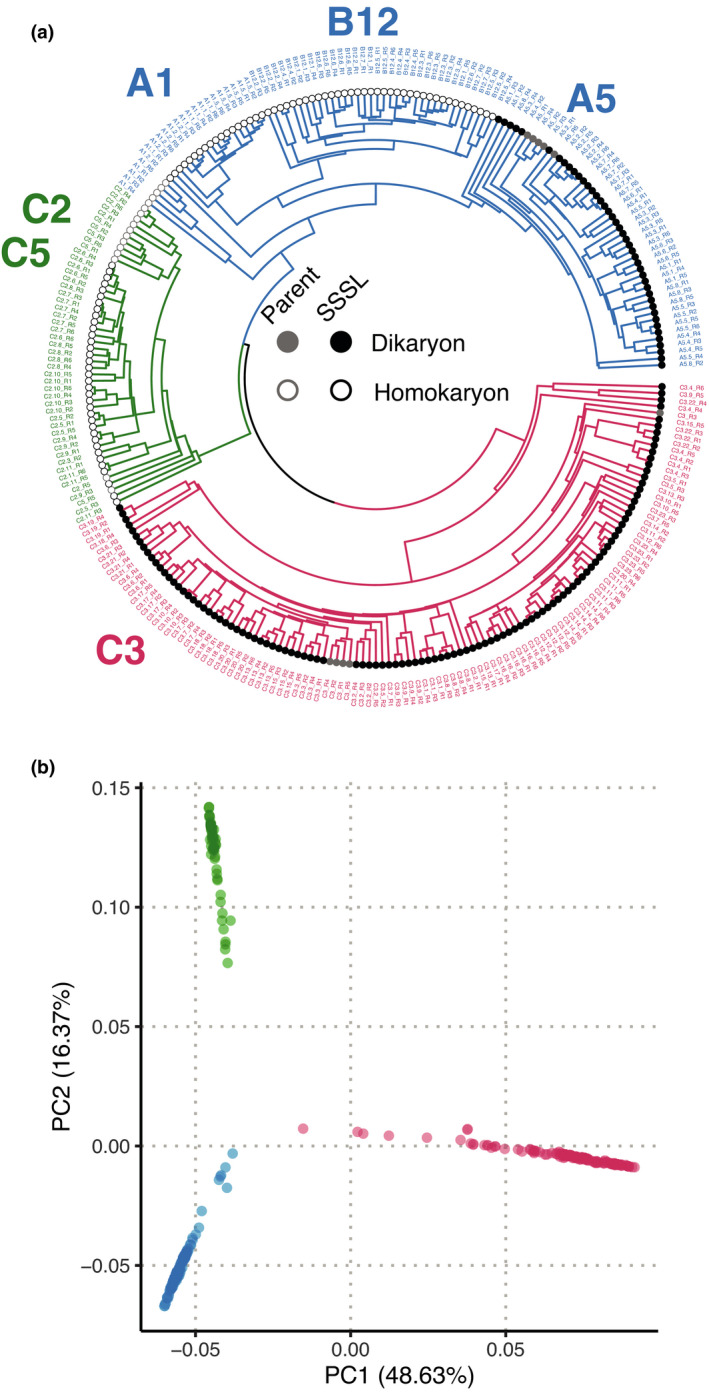
Qualitative analysis of 165 303 biallelic sites in *Rhizophagus* *irregularis* parental isolates and their 48 single‐spore sibling lines (SSSLs). (a) Relationship between parents and SSSL progeny based on shared polymorphisms. Homokaryon parents and SSSLs are labelled with grey and black open circles, respectively, and dikaryon parents and SSSLs are labelled with grey and black filled circles, respectively. (b) Principal component analysis of present and absent polymorphisms showing distinct clustering of three groups. Colours follow the groupings shown in (a). ddRADseq, double‐digest restriction‐site‐associated DNA sequencing; RNAseq, RNA sequencing.

We detected 165 303 polymorphic loci in the ddRADseq data from *R. irregularis* parental isolates and all 48 SSSLs. These polymorphic loci allowed us to infer qualitative similarity among the parents and their SSSLs. The relationship among parental isolates conformed to that expected from previous results (Wyss *et al*., 2016; Savary *et al*., 2018a; Masclaux *et al*., 2019). We recovered three distinct groups: a group with C3 and its offspring, a second group with C2, C5, and their offspring, and a third group comprising A1, B12, and A5 and their offspring (Fig. 2a). Parents and their SSSLs showed a clear separation among three distinct clusters based on the presence and absence of multiple polymorphic sites (Fig. 2b). All SSSLs clustered similarly with their parent and were, therefore, considered qualitatively indistinguishable, as would be expected for offspring from clonal reproduction.

### Quantitative assessment of biallelic sites in *Rhizophagus irregularis* parental isolates and their 48 single‐spore sibling lines

**Fig. 3:**
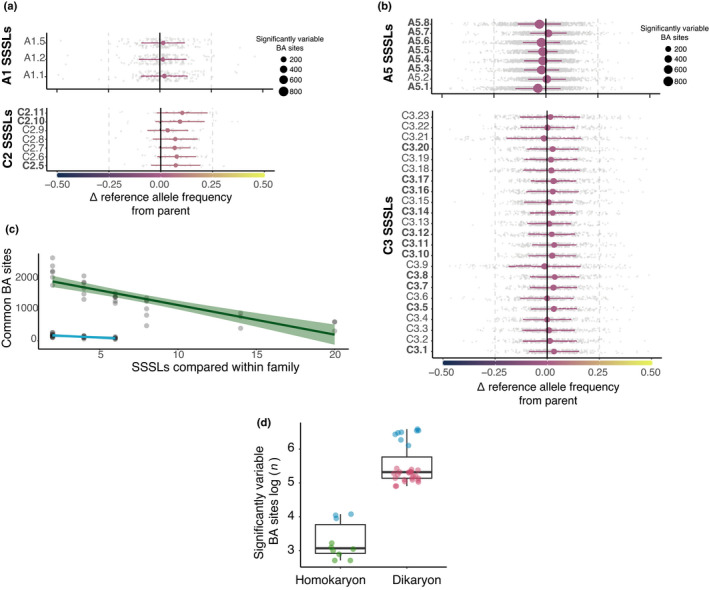
Quantitative changes in allele frequencies at biallelic (BA) sites between *Rhizophagus* *irregularis* parental isolates and their single‐spore sibling lines (SSSLs). (a) Variation in reference allele (RA) frequencies among homokaryon SSSLs and their respective parent A1 and C2. The difference in RA frequency at each common BA site of a given sibling from its parent is plotted as a grey dot. The mean change in the RA frequency of an SSSL from its parent (and the SD) are plotted, with the point size indicating the number of significantly different BA sites (chi‐squared, *P* < 0.05) The colour of the point indicates an increase or decrease in the RA compared with the parent. Significant overall shifts (*t*‐test, *P* < 0.05) in SSSLs from parents are represented as bolded SSSL labels on the *y*‐axis. Significant shifts (*t*‐test, *P* < 0.05) in allele frequencies of SSSLs are represented as bolded labels of SSSLs on the *y*‐axis. (b) Variation in RA frequencies between dikaryon SSSLs and their respective parent A5 and C3. Analysis and plots follow the same format as in (a). (c) Regression analysis of family (SSSLs originating from a parent) size effects on commonly detected BA sites. Homokaryons (light blue; adjusted *R* ^2^ = 0.3773, *P = *0.0014) and dikaryons (green; adjusted *R* ^2^ *=* 0.6964, *P < *0.0001). Green shading represents the 95% interval of confidence of the regression line. (d) Number of biallelic sites at which significant variation was found in allele frequencies in homokaryon and dikaryon *R. irregularis* families. Box‐and‐whisker plots show the distribution of significant BA sites for each SSSL. The scaled log values of the number of BA sites differing significantly in allele frequency between the parent and an SSSL (chi‐squared, *P < *0.05) is represented on the *y*‐axis. The number of significantly variable loci between a parent and progeny is significantly higher in dikaryons (*t* = −13.088, df = 9, *P* = 3.665 × 10^−7^; one‐sided *t*‐test). Green dots represent C2, C5, and SSSLs of C2. Blue dots in the homokaryon box represent A1 and its SSSLs. Red dots represent C3 and its SSSLs. The horizonal line represents the median, the box represents the interquartile range, and the vertical lines represent the maximum and minimum values.

Very few biallelic sites were shared among homokaryon parents A1 and C2 and their SSSLs (167 and 32 sites, respectively; Fig. 3a). By contrast, dikaryon isolates A5 and C3 shared more biallelic sites with their progeny (1233 and 299, respectively; Fig. 3b). We tested whether this difference between dikaryons A5 and C3 was influenced by the high number of C3 SSSLs being compared. Indeed, we detected fewer common biallelic sites in dikaryons as we considered more SSSLs (Fig. 3c). There was a significant negative correlation between commonly detected biallelic sites and the number of SSSLs compared (−0.6379, *P* = 0.0014 in homokaryons; −0.8408, *P* < 0.001 in dikaryons).

We tested whether reference allele frequencies at common biallelic sites increased or decreased significantly (i.e. quantitatively varied) in SSSLs relative to the frequencies in their parent. By subjecting read counts at biallelic sites to a chi‐squared test, we found significant differences in relative allele frequencies between a parent and its offspring in both homokaryons and dikaryons (15–59 sites and 135–727 sites, respectively; Fig. 3a,b). The reference allele frequency differences between a parent and their SSSLs were significantly higher in dikaryons than in homokaryons (Fig. 3a,b,d).

We analysed mean reference allele frequencies at all significant biallelic sites between a parent and SSSL to understand whether these sites resulted in salient increases or decreases in reference allele frequencies of a given SSSL (Fig. 3a,b). We found that homokaryon SSSLs generally displayed fewer significant shifts in their reference allele frequency than their parent did, and several experienced no significant change (Fig. 3a). Still, some differences were observed in some homokaryon SSSLs of C2, but these may represent stochastic variation or positions at which there are potential problems in the genome assembly (Masclaux *et al*., 2019). More striking were changes in reference allele frequency occurring in dikaryon SSSLs; notably, that changes were bidirectional, representing both reference allele increases and decreases in SSSLs compared with their parent (Fig. 3b). Most of the A5 SSSLs retained a lower reference allele frequency than the parent did (Fig. 3b). Only SSSL A5.7 showed a significant increase in the reference allele frequency compared with A5. There was a much broader range of variation among SSSLs of C3. The SSSLs C3.1, C3.5, C3.7, C3.8, C3.10, C3.11, C3.12 C3.14, C3.16, C3.17 and C3.20 all exhibited highly significant reference allele frequency increases compared with C3. The SSSLs C3.3, C3.6, C3.13, C3.15, C3.19 and C3.22 all showed similar reference allele frequencies to C3, and SSSLs C3.4, C3.9 and C3.21 showed decreases in the reference allele frequency compared with the parent C3.

### Concordance of biallelic sites in double‐digest restriction‐site‐associated DNA‐sequencing and RNA‐sequencing data from single‐spore sibling lines of C3

We observed a large number of shared biallelic sites in the genome among SSSL replicates, ranging from 1740 to 2318 in C3.6 and C3.8, respectively (Fig. S1). All SSSLs shared 1409 common genomic biallelic sites, with 684 located in coding regions (Fig. S2a,b). Of the 684 genomic biallelic sites observed in coding regions in ddRADseq data, only some of these were observed in the transcriptome, ranging from 130 to 144 in C3.6 and C3.21, respectively (Fig. S2c). RNAseq reproducibility was lower than that observed in ddRADseq data, as many variants were unique to one technical replicate, being most likely sequencing artefacts due to the large differences in sequencing depth between the two experiments (Fig. S3; Tables S4, S5). Despite this, thousands of biallelic sites were consistent among replicates, and ranging from 5989 to 7117 in C3.8 and C3.21, respectively. Conservative posterior analyses of allele frequencies were restricted to a subset of these biallelic sites, resulting in from 479 to 757 (in C3.6 and C3.7, respectively; ddRADseq) and from 728 to 1445 biallelic sites (in C3.9 and C3.7, respectively; RNAseq).

### Variation in allele frequencies among five dikaryon single‐spore sibling lines of C3

**Fig. 4:**
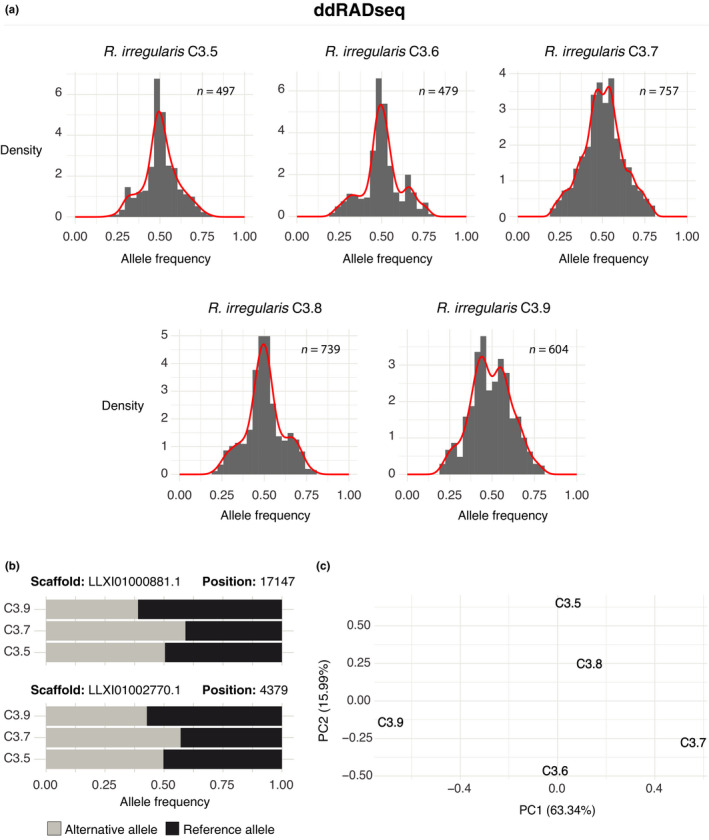
Analysis of double‐digest restriction‐site‐associated DNA sequencing (ddRADseq) data of five dikaryon single‐spore sibling lines (SSSLs) of C3. (a) Distribution of allele frequencies of biallelic (BA) sites in *Rhizophagus* *irregularis* SSSLs. (b) Two examples of BA sites showing differences in their relative proportions between the two siblings C3.7 and C3.9. (c) Principal component analysis of allele frequencies at BA sites. Score plot based on the allele frequencies of common BA sites of the SSSLs (*n* = 577 sites). The first two principal components are shown with their respective percentages of explained variance.

Allele frequency distributions of biallelic sites displayed the expected peaks of the diploid (0.5) and tetraploid controls (0.25; 0.5; 0.75) (Fig. S4). Similarly, we examined allele frequencies among all SSSLs at common biallelic sites in ddRADseq data and revealed that SSSLs C3.5 (497 sites), C3.6 (479 sites), and C3.8 (739 sites) exhibited a unimodal allele frequency distribution centred at 0.5 (Fig. 4a). By contrast, two other SSSLs, C3.7 (757 sites) and C3.9 (604 sites), displayed bimodal distributions with peaks around 0.45 and 0.55 in C3.7 and 0.40 and 0.60 in C3.9 (Fig. 4a). Furthermore, even though both these SSSLs displayed bimodal distributions, reference allele frequencies were opposing. More specifically, at a given site, the reference allele frequency was higher in C3.9 and the alternative allele frequency was higher in C3.7 (Fig. 4b). Using allele frequencies at 125 common biallelic sites, PCA revealed SSSL dissimilarity at these biallelic sites, explaining 63.3% (PC1) of the variance (Fig. 4c). We observed that SSSLs were distributed along PC1, likely representing the variation in abundance of nuclear genotypes among SSSLs. The three dikaryon SSSLs (C3.5, C3.6 and C3.8) that showed a 1 : 1 ratio of both nuclei (unimodal distributions) clustered together at the centre. The two dikaryon strains (C3.7 and C3.9) that displayed unequal proportions (bimodal distributions) of allele frequencies were diametrically opposed along PC1 (Fig. 4c).

### Transcriptome‐wide differences in allele expression among six dikaryon single‐spore sibling lines of C3

**Fig. 5:**
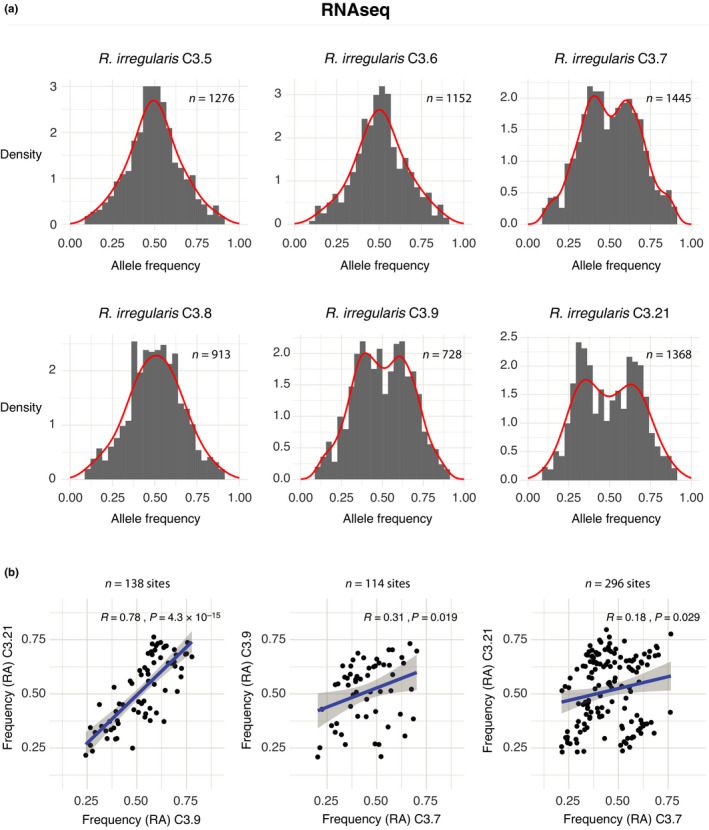
Analysis of allele frequencies on RNA sequencing (RNAseq) data. (a) Distribution of allele frequencies of biallelic (BA) sites of six dikaryon single‐spore sibling lines of C3. (b) Pairwise comparisons of allele frequencies of common BA sites between *Rhizophagus* *irregularis* strains C3.7, C3.9, and C3.21. Correlation of the allele frequencies (reference allele, RA) at common BA sites (Pearson’s correlation coefficient and probability are shown). Grey shading represents the 95% interval of confidence of the regression line.

We then addressed whether RNAseq data revealed transcriptional bias at biallelic sites in dikaryon SSSLs. A remarkably similar allele frequency distribution to that observed in the genomic data also occurred in SSSL transcriptomes (Fig. 5a). Similar to ddRADseq, biallelic sites in SSSLs C3.5 (1276 sites), C3.6 (1152 sites), and C3.8 (913 sites) showed unimodal distributions centred at 0.5. SSSLs C3.7 (1445 sites) and C3.9 (728 sites) again presented clear bimodal distributions in their transcript frequencies, similar to unequal allele frequencies observed in ddRADseq. C3.21 (1368 sites) also exhibited a bimodal distribution, with the most extreme allele frequencies transcribed of all SSSLs (an approximate 3 : 7 ratio).

We compared transcript allele frequencies at common biallelic sites to determine similarity among SSSLs with bimodal distributions. Pairwise comparisons between C3.9 and C3.21 allele frequencies revealed a positive correlation (*R* = 0.78) and a transcription bias towards the same, most abundant allele (Fig. 5b). By contrast, pairwise comparison of C3.7 with C3.9 (*R* = 0.31) and C3.7 with C3.21 (*R* = 0.18) showed much weaker correlation. This result is congruent with observations of ddRADseq data, where reference allele frequencies of SSSLs C3.7 and C3.9 were opposing.

### Genes under allelic imbalance during transcription

**Fig. 6:**
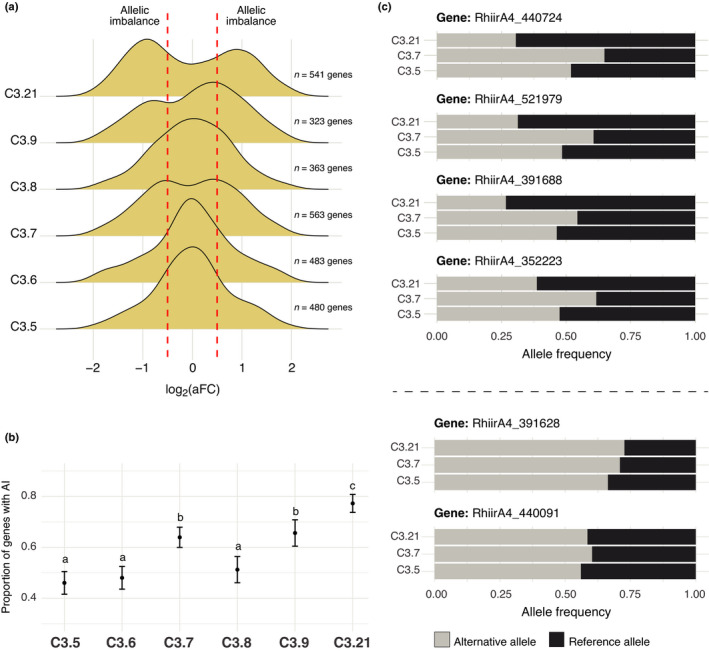
Allelic imbalance (AI) during gene transcription among six dikaryon single spore sibling lines (SSSLs) of C3. (a) Distribution of the transcriptional allelic fold‐change (aFC) of biallelic (BA) genes in the six *Rhizophagus* *irregularis* SSSLs. Red‐dotted lines represent the thresholds for a significant AI. (b) Proportion of genes under a significant AI. (c) Four examples of genes revealing opposing patterns of AI in *R. irregularis* C3.7 and C3.21 (top); and two examples of genes that did not show differences in allele expression among SSSLs (bottom).

Similar to ddRADseq, and global RNAseq analyses, we further observed bimodal distributions in SSSLs C3.7, C3.9, and C3.21 when testing for allelic fold‐change variation in gene transcripts based on one biallelic site (Fig. 6a). Genes exhibiting allelic imbalance were present in all six SSSLs, even though allele frequency distributions centred at 0.5 (Table S8). Still, allelic imbalance of biallelic expressed genes significantly differed among the SSSLs (*χ* ^2^ = 152.71, df = 5, *P* < 2.2 × 10^−16^) and, indeed, was more pronounced in SSSLs with bimodal allele frequency distributions. For example, C3.21 showed the highest proportion of genes under allelic imbalance (close to 80%) and a slightly lower proportion in C3.7 and C3.9 (60–70%) (Fig. 6b; Table S9). Allele frequencies of several genes differed by up to *c*. 25% between SSSLs C3.7 and C3.21 and were consistently dissonant (Fig. 6c, top). Other biallelic expressed genes exhibited similar allele frequencies among the SSSLs (Fig. 6c, bottom). Notably, most genes under allelic imbalance were unique to individual SSSLs (Fig. S5a) and possessed a wide variety of biological functions (Fig. S5b).

### Monoallelic expression of biallelic sites within genes

**Fig. 7:**
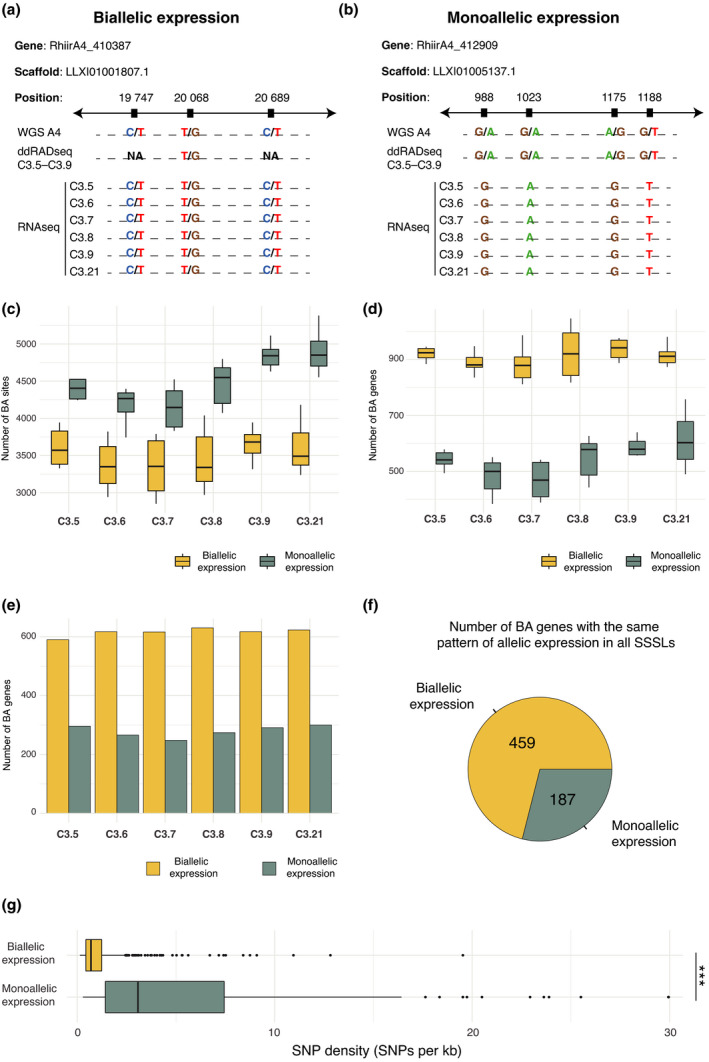
Expression of biallelic (BA) sites in the transcriptomes of six single‐spore sibling lines (SSSLs) of C3. (a) Example of a BA gene that exhibited BA expression in all SSSLs. ddRADseq, double‐digest restriction‐site‐associated DNA sequencing; RNAseq, RNA sequencing. (b) Example of a BA gene that exhibited monoallelic (MA) expression of its BA sites in all SSSLs. (c) Number of BA sites where both alleles were expressed (BA expression) and number of BA sites where only one allele was expressed (MA expression). The horizonal line represents the median, the box represents the interquartile range, and the vertical lines represent the maximum and minimum values. (d) Number of BA genes that show BA expression or MA expression. The horizonal line represents the median, the box represents the interquartile range, and the vertical lines represent the maximum and minimum values. (e) Number of BA genes with BA expression or MA expression that were consistent among all replicates of each SSSL. (f) Number of BA genes that showed BA expression or MA expression consistently among all replicates of all SSSLs. (g) Density of polymorphic sites (single‐nucleotide polymorphisms (SNPs) per kilobase) in genes that exhibited BA expression and MA expression. The horizonal line represents the median, the box represents the interquartile range, and the vertical lines represent the maximum and minimum values.

RNAseq data at biallelic sites revealed that either both alleles or sometimes only one allele was transcribed (Fig. 7a,b). We, therefore, further investigated the prevalence of monoallelic expression at biallelic sites and found that the number of biallelic sites with biallelic expression was lower than those with monoallelic expression (Fig. 7c). Still, biallelic expressed genes were significantly higher than monoallelic expressed genes in all SSSLs (Fig. 7d). Approx. 600 biallelic expressed and 250 monoallelic expressed genes were identified in each SSSL, of which 459 and 187, respectively, were commonly shared among all six SSSLs (Fig. 7e,f). Most notably, monoallelic expressed genes had significantly higher SNP densities, compared with biallelic expressed genes (Fig. 7g), and were significantly less expressed than biallelic expressed genes (Fig. S6). In both cases, the functional annotation of genes with monoallelic expression and biallelic expression revealed orthologues involved in many, and sometimes common, biological processes, such as energy production and conversion, transcription, or signal transduction mechanisms (Tables S10, S11).

## Discussion

In this study, we generated ddRADseq data on a cohort of 48 homokaryon and dikaryon SSSLs of their *R. irregularis* parental isolates. We showed that SSSLs are indeed clonal offspring, but that dikaryon SSSLs, despite qualitatively being clones, commonly exhibit quantitative allele frequency variation at biallelic sites. This variation represents proportions of two genetically distinct nuclei. Analysis on a subsample of dikaryon SSSLs from one parent revealed that the frequency of two nuclear genotypes deviated considerably from the parent. Ultimately, this translated into the predominance of one of the two nuclear genotypes in some SSSLs. Both nuclear genotypes contributed to gene transcription, and the transcription of biallelic genes mirrored nuclear genotype frequencies. Monoallelic expression also sometimes occurred in genes that were biallelic, and this was more likely if there was a greater divergence between alleles (i.e. a higher SNP per kilobase density) of the gene.

### *Rhizophagus irregularis* dikaryons produce clonal single‐spore sibling lines that quantitatively differ in nuclear genotype proportions

**Fig. 8:**
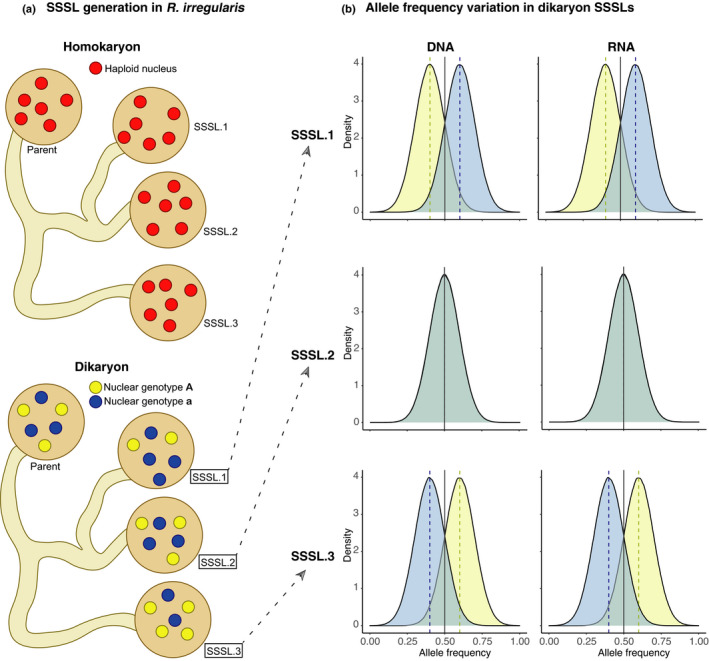
A schematic diagram summarizing the relationship between observed proportions of nuclear genotypes in *Rhizophagus* *irregularis* parents and offspring and their gene transcription patterns. (a) A parental homokaryon isolate gives rise to clonally identical offspring (shown as red nuclei). A dikaryon parental isolate gives rise to offspring with relative proportions of two nuclear genotypes that can diverge in a single‐spore sibling line (SSSL) from the parental isolate (e.g. SSSL.1 and SSSL.3). The nuclei of the two different genotypes are shown in yellow and blue. These proportions represent allele frequencies estimations in double‐digest restriction‐site‐associated DNA sequencing data. (b) Allele frequencies at biallelic sites in the genome and in the transcriptome of the three SSSLs 1, 2 and 3 shown in (a). Colours under the allele frequency curves represent alleles originating from each of the two nuclear genotypes.

Using ddRADseq data, we analysed more SSSLs than previous studies, and many more than would be possible with single nuclei sequencing. Multiple loci enabled us to assess genetic variation in homokaryon and dikaryon SSSLs (Fig. 8a). SSSLs clustered with their parents, indicating no significantly detectable qualitative genetic variation. Although a small number of biallelic sites were still detected in homokaryons, we expect that they have little to no functional consequence (Masclaux *et al*., 2019). Although the lack of genetic variation among homokaryon SSSLs is intuitive, it is interesting in the context of recent field‐based experiments. Large significant differences in cassava yield were observed in the field in a fully replicated randomized‐block design experiment when cassava was inoculated with SSSLs originating from homokaryon parents (Ceballos *et al*., 2019; Peña *et al*., 2020). The SSSLs were the same ones on which ddRADseq was performed in this study. It is, therefore, improbable that yield differences induced by inoculation with different SSSLs can be attributed to quantitative genetic variation among homokaryon SSSLs, and so likely depends on additional, contextual factors, including potential epigenetic differences among SSSLs and how SSSLs affect soil microbial community composition and succession (Gao *et al*., 2019).

In contrast to homokaryons, we observed much more quantitative variation among dikaryon SSSLs. As hypothesized, biallelic sites were more prolific among dikaryon SSSLs and reflect the presence of two genetically distinct nuclei. Furthermore, reference allele frequencies at multiple biallelic sites quantitatively deviated between clonal SSSLs and their parent, indicating the inheritance of different nuclear genotype proportions.

Typical allele frequency distributions at biallelic sites in diploid organisms are unimodally distributed and centred at 0.5 (Zhu *et al*., 2016) (Fig S4). Similarly, AMF isolates with a population of two distinct haploid nuclear genotypes (e.g. a dikaryon) should display diploid‐like allele frequency distributions (Ropars *et al*., 2016). On the other hand, disproportionate inheritance of nuclei would result in deviations from 0.5 (Masclaux *et al*., 2018). We provide additional support based on more detailed analyses that SSSLs of C3 varied between 2 : 3 and 3 : 2 in nuclear ratios from their parent (1 : 1; alternative : reference allele frequency). Specifically, our results strongly indicate disproportionate inheritance of two nuclear genotypes in C3.7 and C3.9 and that quantitative genetic variation often occurs among dikaryon SSSLs (Fig. 8a). A previous single‐locus study of *bg112* allele frequencies arrived at a similar conclusion but, despite adequate replication, was scrutinized due to possible PCR variability (Masclaux *et al*., 2018; Kokkoris *et al*., 2020). We confirm earlier results and can conclude that this criticism is highly unlikely, given that independent and well‐replicated data sets produced near‐identical results across hundreds of biallelic sites. An alternative explanation for quantitative genetic differences observed among siblings would be that nuclei fused and recombined. However, this is unlikely. Single‐nucleus sequencing of *R. irregularis* isolate A4 (which is genetically indistinguishable from C3 and is, thus, considered a clone) revealed no evidence (Chen *et al*., 2018a) of among‐nucleus recombination in this fungus. Although the same study detected a very small amount of recombination among nuclei of another isolate of the same species, this remains controversial (Auxier & Bazzicalupo, 2019).

### Allelic imbalance in gene transcription in *Rhizophagus irregularis* dikaryons

Previously, researchers suggested that gene expression in a dikaryon isolate might reflect proportions of both nuclei (Masclaux *et al*., 2018). Because some SSSLs of C3 displayed different nuclear genotype proportions, we wanted to see if evidence of the same could be found in transcriptome profiles of these SSSLs. The results confirmed that SSSLs with disproportionate nuclear genotypes based on ddRADseq data also displayed allelic imbalance in biallelic transcripts, reflecting allele frequencies found in ddRADseq (Fig. 8b). These observations suggest a direct consequence of unequal nuclear genotype ratios on transcribed alleles, with the most abundant genotype being transcriptionally overrepresented. This is an important result because this indicates that the generation of such quantitative genetic variation could also potentially influence the AMF phenotype. Furthermore, because a previous study has shown associations between patterns of genome variation in *R. irregularis* and plant growth, such alterations in nuclear genotype frequency could potentially influence the symbiosis with plants (Ceballos *et al*., 2019).

### Exceptions to the rule: when transcribed alleles do not reflect nuclear genotype ratios

Allele frequencies of transcripts did not always reflect the estimated nuclear genotype ratios, but this represented a much smaller number of biallelic genes than those that were expressed in the same proportion as the nuclear genotype frequencies. Interestingly, some biallelic genes exhibited the same pattern of allelic imbalance in expression in all SSSLs, regardless of nuclei proportions (Fig. 6c). Therefore, it is likely that some genes are affected by other transcriptional regulatory mechanisms that are independent of nuclear genotype proportions.

### Biallelic vs monoallelic expression at biallelic sites suggests multilayered regulation of transcription in dikaryons

We observed that at many biallelic sites in the genome only one of the two possible sequence variants was actually transcribed. This was a consistent and significant pattern across all six SSSLs, irrespective of nuclear genotype ratios (Fig. 7). Intriguingly, significantly more biallelic genes expressed both alleles, rather than showing monoallelic expression. Again, this was a remarkably similar pattern across all SSSLs and across replicates of each SSSL, revealing a very robust pattern (Fig. 7). Taken together, these results show that biallelic genes in which greater divergence between the two alleles has occurred (as measured by the number of biallelic sites in the gene) are less likely to both be transcribed. These results point both to biased monoallelic expression at highly polymorphic sites and to possible epigenetic silencing of highly divergent alleles, a situation that is predicted in a conflictual scenario between two divergent genomes (Dyson & Goodisman, 2020; Zou *et al*., 2020).

One other completely unexpected result was that transcription was also consistently significantly higher in biallelic expressed genes than in monoallelic expressed genes across all replicates and all SSSLs (Fig. S6). The fact that transcription of both alleles of a gene gave rise to significantly more transcripts than those exhibiting monoallelic expression suggests that, in more highly divergent biallelic genes, suppression of one allele limits the transcription of the gene. A prediction from this finding would be that genes that are required to respond to a sudden environmental cue by rapidly producing a high transcript number should be under selection to retain two alleles that have undergone little divergence. However, we cannot completely exclude the possibility that overall lower expression levels of monoallelic expressed genes hindered the detection of the second allele in some cases.

It was important that all cultures were maintained in a homogeneous environment, so as not to influence transcription results. In cases where monoallelic expression occurs in a biallelic gene, it is also possible that selection would favour the retention of two divergent alleles that could be differentially expressed in different environments. Experimentally manipulated environments may shed light on this possibility.

### Ecological significance and application of quantitative variation among dikaryon single‐spore sibling lines

Fungi typically display an array of nuclear dynamics to fit their life strategies. For example, yeasts, which are not host dependent, show a fitness cost associated with being diploid; consequently, haploid strains adapt and evolve much faster (Marad *et al*., 2018). On the other hand, the obligate plant pathogenic rust fungus *Puccinia graminis* f. sp. *trici* needs two plant hosts to complete its life cycle, but it can only infect alternate hosts with homokaryon spores and primary hosts with dikaryon spores (Bakkeren & Szabo, 2020). Scott *et al*. (2019) recently compared two models of AMF evolution in which selection acts either on individuals or on the nucleus. The current opinion is that high intraspecific genetic diversity in *R. irregularis* could facilitate evolution by enabling generalist lifestyles and overcoming the danger of becoming too specialized on one host (Chen *et al*., 2018b). This relationship was explored and demonstrates that nuclear dynamics may change in response to particular plant hosts for dikaryon AMF (Angelard *et al*., 2014; Kokkoris *et al*., 2021). In nature, AMF dikaryons may optimize niche adaptation in multiple ecosystems by maintaining both populations of cooperating nuclear genotypes. Dikaryon SSSLs indeed exhibit large differences in quantitative traits and affect plant growth significantly (Angelard *et al*., 2010; Ceballos *et al*., 2013, 2019; Peña *et al*., 2020). This might perhaps be due to the fact that SSSLs with the most optimal ratios of nuclear genotypes colonize and form symbioses with a given host more rapidly.

In conclusion, we show that dikaryon *R. irregularis* isolates commonly generate quantitative shifts in allele frequencies among single‐spore offspring. These shifts in allele frequencies are observed in hundreds of biallelic sites across the genome and likely reflect the changes in proportions of the two nuclear genotypes. We further conclude that varying nuclear dynamics of SSSLs generate similar quantitative shifts in gene transcription, meaning that transcription is linked to the underlying nuclear ratios of SSSLs. These findings hint toward additional factors to consider that may regulate transcription and symbiosis within these important plant mutualists.

## Author contributions

CR, JCC and IRS designed the experiments; CR, CA and RS produced the ddRADseq data on 48 SSSLs; JCC, CA, S‐JL and RS produced the RNAseq data on the six dikaryon SSSLs; FGM generated the ddRADseq on the five dikaryon SSSLs; CR and JCC analysed sequencing data; CR, JCC, S‐JL, IDM and IRS interpreted data and wrote the manuscript; IRS acquired project funding. CR and JCC contributed equally to this work.

## Supporting information

**Fig. S1** Reproducibility of bi‐allelic (BA) sites in the ddRAD‐sequencing data.
**Fig. S2** Consistency of bi‐allelic (BA) sites in ddRADseq data and their transcription.
**Fig. S3** Reproducibility of bi‐allelic (BA) sites in the RNA‐sequencing data.
**Fig. S4** Allele frequency distributions of diploid (*C. albicans* and *B. nana*) and tetraploid (*B.* × *intermedia*) species derived from ddRAD‐seq data.
**Fig. S5** Genes with allele transcription under allelic imbalance.
**Fig. S6** Reproducibility of mono‐allelic and bi‐allelic expression among three biological replicates of each of the 6 dikaryon single spores sibling lines (SSSLs) of *R. irregularis* C3.

**Table S1** List of all primer sequences used during ddRADseq library preparation.
**Table S2** Summary of the ddRAD‐sequencing mapping statistics on 48 single spores sibling lines (SSSLs).
**Table S3** Results of chi‐squared and Mann‐Whitney‐U statistical tests for quantitative variance at bi‐allelic sites.
**Table S4** Mapping statistics of ddRADseq sequences from 5 dikaryon single spores sibling lines of C3.
**Table S5** Summary of the RNA‐sequencing mapping statistics from 6 dikaryon single spores sibling lines of C3.
**Table S6** Coverage statistics of bi‐allelic sites detected in ddRADseq data.
**Table S7** Coverage statistics of bi‐allelic sites detected in RNAseq data.
**Table S8** List of genes under allelic imbalance.
**Table S9** Post‐hoc tests for the pairwise comparison of the proportions of studied genes found under allelic imbalance.
**Table S10** Functional assessment bi‐allelic expressed genes.
**Table S11** Functional assessment of mono‐allelic expressed genes.
Please note: Wiley Blackwell are not responsible for the content or functionality of any Supporting Information supplied by the authors. Any queries (other than missing material) should be directed to the *New Phytologist* Central Office.

## Data Availability

All sequencing data are deposited in the European Nucleotide Archive under the following accession nos.: PRJEB37069, PRJEB39082, PRJEB39188.

## References

[nph17530-bib-0001] Angelard C, Colard A, Niculita‐Hirzel H, Croll D, Sanders IR. 2010. Segregation in a mycorrhizal fungus alters rice growth and symbiosis‐specific gene transcription. Current Biology 20: 1216–1221. 20541408 10.1016/j.cub.2010.05.031

[nph17530-bib-0002] Angelard C, Tanner CJ, Fontanillas P, Niculita‐Hirzel H, Masclaux F, Sanders IR. 2014. Rapid genotypic change and plasticity in arbuscular mycorrhizal fungi is caused by a host shift and enhanced by segregation. ISME Journal 8: 284–294. 10.1038/ismej.2013.154 PMC3906815 24030596

[nph17530-bib-0003] Auxier B, Bazzicalupo A. 2019. Comment on 'Single nucleus sequencing reveals evidence of inter‐nucleus recombination in arbuscular mycorrhizal fungi'. eLife 8: e47301. 31650958 10.7554/eLife.47301 PMC6814362

[nph17530-bib-0004] Bago B, Pfeffer PE, Shachar‐Hill Y. 2000. Carbon metabolism and transport in arbuscular mycorrhizas. Plant Physiology 124: 949–958. 11080273 10.1104/pp.124.3.949 PMC1539291

[nph17530-bib-0005] Bakkeren G, Szabo LJ. 2020. Progress on molecular genetics and manipulation of rust fungi. Phytopathology 110: 532–543. 31799902 10.1094/PHYTO-07-19-0228-IA

[nph17530-bib-0006] Bolger AM, Lohse M, Usadel B. 2014. Trimmomatic: a flexible trimmer for Illumina sequence data. Bioinformatics 30: 2114–2120. 24695404 10.1093/bioinformatics/btu170 PMC4103590

[nph17530-bib-0007] Bravo A, Brands M, Wewer V, Dormann P, Harrison MJ. 2017. Arbuscular mycorrhiza‐specific enzymes FatM and RAM2 fine‐tune lipid biosynthesis to promote development of arbuscular mycorrhiza. New Phytologist 214: 1631–1645. 10.1111/nph.14533 28380681

[nph17530-bib-0008] Brundrett MC, Tedersoo L. 2018. Evolutionary history of mycorrhizal symbioses and global host plant diversity. New Phytologist 220: 1108–1115. 10.1111/nph.14976 29355963

[nph17530-bib-0009] Catchen JM, Amores A, Hohenlohe P, Cresko W, Postlethwait JH. 2011. Stacks: building and genotyping loci *de novo* from short‐read sequences. Genes Genomes Genetics 1: 171–182. 22384329 10.1534/g3.111.000240 PMC3276136

[nph17530-bib-0010] Ceballos I, Mateus‐Gonzalez ID, Peña R, Peña‐Quemba DC, Robbins C, Ordoñez YM, Rosikiewicz P, Rojas EC, Thuita M, Mlay DP*et al*. 2019. Using variation in arbuscular mycorrhizal fungi to drive the productivity of the food security crop cassava. *bioRxiv*. doi: 10.1101/830547.

[nph17530-bib-0011] Ceballos I, Ruiz M, Fernandez C, Pena R, Rodriguez A, Sanders IR. 2013. The *in vitro* mass‐produced model mycorrhizal fungus, *Rhizophagus irregularis*, significantly increases yields of the globally important food security crop cassava. PLoS ONE 8: e70633. 23950975 10.1371/journal.pone.0070633 PMC3737348

[nph17530-bib-0012] Chen ECH, Mathieu S, Hoffrichter A, Sedzielewska‐Toro K, Peart M, Pelin A, Ndikumana S, Ropars J, Dreissig S, Fuchs J*et al*. 2018a. Single nucleus sequencing reveals evidence of inter‐nucleus recombination in arbuscular mycorrhizal fungi. eLife 7: e39813. 30516133 10.7554/eLife.39813 PMC6281316

[nph17530-bib-0013] Chen ECH, Morin E, Beaudet D, Noel J, Yildirir G, Ndikumana S, Charron P, St‐Onge C, Giorgi J, Kruger M*et al*. 2018b. High intraspecific genome diversity in the model arbuscular mycorrhizal symbiont *Rhizophagus irregularis* . New Phytologist 220: 1161–1171. 10.1111/nph.14989 29355972

[nph17530-bib-0014] Clergeot PH, Rode NO, Glémin S, Brandström Durling M, Ihrmark K, Olson Å. 2019. Estimating the fitness effect of deleterious mutations during the two phases of the life cycle: a new method applied to the root‐rot fungus *Heterobasidion parviporum* . Genetics 211: 963–976. 30598467 10.1534/genetics.118.301855 PMC6404244

[nph17530-bib-0015] Croll D, Giovannetti M, Koch AM, Sbrana C, Ehinger M, Lammers PJ, Sanders IR. 2009. Nonself vegetative fusion and genetic exchange in the arbuscular mycorrhizal fungus *Glomus intraradices* . New Phytologist 181: 924–937. 10.1111/j.1469-8137.2008.02726.x 19140939

[nph17530-bib-0016] Davison J, Moora M, Opik M, Adholeya A, Ainsaar L, Ba A, Burla S, Diedhiou Ag, Hiiesalu I, Jairus T*et al*. 2015. Global assessment of arbuscular mycorrhizal fungus diversity reveals very low endemism. Science 349: 970–973. 26315436 10.1126/science.aab1161

[nph17530-bib-0017] Dobin A, Davis CA, Schlesinger F, Drenkow J, Zaleski C, Jha S, Batut P, Chaisson M, Gingeras TR. 2013. Star: ultrafast universal RNA‐seq aligner. Bioinformatics 29: 15–21. 23104886 10.1093/bioinformatics/bts635 PMC3530905

[nph17530-bib-0018] Dong X, Zhang M, Chen J, Peng L, Zhang N, Wang X, Lai J. 2017. Dynamic and antagonistic allele‐specific epigenetic modifications controlling the expression of imprinted genes in maize endosperm. Molecular Plant 10: 442–455. 27793787 10.1016/j.molp.2016.10.007

[nph17530-bib-0019] Dyson CJ, Goodisman MAD. 2020. Gene duplication in the honeybee: patterns of DNA methylation, gene expression, and genomic environment. Molecular Biology and Evolution 37: 2322–2331. 32243528 10.1093/molbev/msaa088

[nph17530-bib-0020] Ehinger MO, Croll D, Koch AM, Sanders IR. 2012. Significant genetic and phenotypic changes arising from clonal growth of a single spore of an arbuscular mycorrhizal fungus over multiple generations. New Phytologist 196: 853–861. 10.1111/j.1469-8137.2012.04278.x 22931497

[nph17530-bib-0021] Fellbaum CR, Gachomo EW, Beesetty Y, Choudhari S, Strahan GD, Pfeffer PE, Kiers ET, Bucking H. 2012. Carbon availability triggers fungal nitrogen uptake and transport in arbuscular mycorrhizal symbiosis. Proceedings of the National Academy of Sciences, USA 109: 2666–2671. 10.1073/pnas.1118650109 PMC3289346 22308426

[nph17530-bib-0022] Gao C, Montoya L, Xu L, Madera M, Hollingsworth J, Purdom E, Hutmacher RB, Dahlberg JA, Coleman‐Derr D, Lemaux PG*et al*. 2019. Strong succession in arbuscular mycorrhizal fungal communities. ISME Journal 13: 214–226. 10.1038/s41396-018-0264-0 PMC6298956 30171254

[nph17530-bib-0023] Garrison E, Marth G. 2012. Haplotype‐based variant detection from short‐read sequencing. arXiv:1207.3907.

[nph17530-bib-0024] Gehrmann T, Pelkmans JF, Ohm RA, Vos AM, Sonnenberg ASM, Baars JJP, Wosten HAB, Reinders MJT, Abeel T. 2018. Nucleus‐specific expression in the multinuclear mushroom‐forming fungus *Agaricus bisporus* reveals different nuclear regulatory programs. Proceedings of the National Academy of Sciences, USA 115: 4429–4434. 10.1073/pnas.1721381115 PMC5924919 29643074

[nph17530-bib-0025] Govindarajulu M, Pfeffer PE, Jin H, Abubaker J, Douds DD, Allen JW, Bücking H, Lammers PJ, Shachar‐Hill Y. 2005. Nitrogen transfer in the arbuscular mycorrhizal symbiosis. Nature 435: 819–823. 15944705 10.1038/nature03610

[nph17530-bib-0026] Huerta‐Cepas J, Szklarczyk D, Heller D, Hernandez‐Plaza A, Forslund SK, Cook H, Mende DR, Letunic I, Rattei T, Jensen LJ*et al*. 2019. eggNOG 5.0: a hierarchical, functionally and phylogenetically annotated orthology resource based on 5090 organisms and 2502 viruses. Nucleic Acids Research 47: D309–D314. 30418610 10.1093/nar/gky1085 PMC6324079

[nph17530-bib-0027] Ihaka R, Gentleman R. 1996. R: a language for data analysis and graphics. Journal of Computational and Graphical Statistics 5: 299–314.

[nph17530-bib-0028] Jensen LJ, Julien P, Kuhn M, von Mering C , Muller J, Doerks T, Bork P. 2008. eggNOG: automated construction and annotation of orthologous groups of genes. Nucleic Acids Research 36: D250–D254. 17942413 10.1093/nar/gkm796 PMC2238944

[nph17530-bib-0029] Keymer A, Pimprikar P, Wewer V, Huber C, Brands M, Bucerius SL, Delaux P‐M, Klingl V, Röpenack‐Lahaye EV, Wang TL*et al*. 2017. Lipid transfer from plants to arbuscular mycorrhiza fungi. eLife 6: e29107. 28726631 10.7554/eLife.29107 PMC5559270

[nph17530-bib-0030] Koch AM, Kuhn G, Fontanillas P, Fumagalli L, Goudet J, Sanders IR. 2004. High genetic variability and low local diversity in a population of arbuscular mycorrhizal fungi. Proceedings of the National Academy of Sciences, USA 101: 2369–2374. 10.1073/pnas.0306441101 PMC356957 14983016

[nph17530-bib-0031] Kokkoris V, Chagnon PL, Yildirir G, Clarke K, Goh D, MacLean AM, Dettman J, Stefani F, Corradi N. 2021. Host identity influences nuclear dynamics in arbuscular mycorrhizal fungi. Current Biology 31: 1531–1538. 33545043 10.1016/j.cub.2021.01.035

[nph17530-bib-0032] Kokkoris V, Stefani F, Dalpé Y, Dettman J, Corradi N. 2020. Nuclear dynamics in the arbuscular mycorrhizal fungi. Trends in Plant Science 25: 765–778. 32534868 10.1016/j.tplants.2020.05.002

[nph17530-bib-0033] Lafon‐Placette C, Hatorangan MR, Steige KA, Cornille A, Lascoux M, Slotte T, Köhler C. 2018. Paternally expressed imprinted genes associate with hybridization barriers in *Capsella* . Nature Plants 4: 352–357. 29808019 10.1038/s41477-018-0161-6

[nph17530-bib-0034] Larsson AJM, Johnsson P, Hagemann‐Jensen M, Hartmanis L, Faridani OR, Reinius B, Segerstolpe Å, Rivera CM, Ren B, Sandberg R. 2019. Genomic encoding of transcriptional burst kinetics. Nature 565: 251–254. 30602787 10.1038/s41586-018-0836-1 PMC7610481

[nph17530-bib-0035] Li H, Durbin R. 2009. Fast and accurate short read alignment with Burrows–Wheeler transform. Bioinformatics 25: 1754–1760. 19451168 10.1093/bioinformatics/btp324 PMC2705234

[nph17530-bib-0036] Li H, Handsaker B, Wysoker A, Fennell T, Ruan J, Homer N, Marth G, Abecasis G, Durbin R, 1000 Genome Project Data Processing Subgroup . 2009. The sequence alignment/map format and SAMtools . Bioinformatics 25: 2078–2079. 19505943 10.1093/bioinformatics/btp352 PMC2723002

[nph17530-bib-0037] Lin K, Limpens E, Zhang Z, Ivanov S, Saunders DGO, Mu D, Pang E, Cao H, Cha H, Lin T*et al*. 2014. Single nucleus genome sequencing reveals high similarity among nuclei of an endomycorrhizal fungus. PLoS Genetics 10: e1004078. 24415955 10.1371/journal.pgen.1004078 PMC3886924

[nph17530-bib-0038] Marad DA, Buskirk SW, Lang GI. 2018. Altered access to beneficial mutations slows adaptation and biases fixed mutations in diploids. Nature Ecology & Evolution 2: 882–889. 29581586 10.1038/s41559-018-0503-9

[nph17530-bib-0039] Masclaux FG, Wyss T, Mateus‐Gonzalez ID, Aletti C, Sanders IR. 2018. Variation in allele frequencies at the *bg112* locus reveals unequal inheritance of nuclei in a dikaryotic isolate of the fungus *Rhizophagus irregularis* . Mycorrhiza 28: 369–377. 29675619 10.1007/s00572-018-0834-z

[nph17530-bib-0040] Masclaux FG, Wyss T, Pagni M, Rosikiewicz P, Sanders IR. 2019. Investigating unexplained genetic variation and its expression in the arbuscular mycorrhizal fungus *Rhizophagus irregularis*: a comparison of whole genome and RAD sequencing data. PLoS ONE 14: e0226497. 31881076 10.1371/journal.pone.0226497 PMC6934306

[nph17530-bib-0041] Mateus ID, Masclaux FG, Aletti C, Rojas EC, Savary R, Dupuis C, Sanders IR. 2019. Dual RNA‐seq reveals large‐scale non‐conserved genotype × genotype‐specific genetic reprograming and molecular crosstalk in the mycorrhizal symbiosis. ISME Journal 13: 1226–1238. 10.1038/s41396-018-0342-3 PMC6474227 30647457

[nph17530-bib-0042] Mateus ID, Rojas EC, Savary R, Dupuis C, Masclaux FG, Aletti C, Sanders IR. 2020. Coexistence of genetically different *Rhizophagus irregularis* isolates induces genes involved in a putative fungal mating response. ISME Journal 14: 2381–2394. 10.1038/s41396-020-0694-3 PMC7490403 32514118

[nph17530-bib-0043] Peña R, Robbins C, Cruz Corella J, Thuita M, Masso C, Vanlauwe B, Signarbieux C, Rodriguez A, Sanders IR. 2020. Genetically different isolates of the arbuscular mycorrhizal fungus *Rhizophagus irregularis* induce differential responses to stress in cassava. Frontiers in Plant Science 11: e596929. 10.3389/fpls.2020.596929 PMC7793890 33424891

[nph17530-bib-0044] Quinlan AR. 2014. BEDtools: the Swiss‐army tool for genome feature analysis. Current Protocols in Bioinformatics 47: 1–34. 25199790 10.1002/0471250953.bi1112s47 PMC4213956

[nph17530-bib-0045] Rodriguez‐Echeverria S, Teixeira H, Correia M, Timoteo S, Heleno R, Opik M, Moora M. 2017. Arbuscular mycorrhizal fungi communities from tropical Africa reveal strong ecological structure. New Phytologist 213: 380–390. 10.1111/nph.14122 27560189

[nph17530-bib-0046] Ropars J, Toro KS, Noel J, Pelin A, Charron P, Farinelli L, Marton T, Krüger M, Fuchs J, Brachmann A*et al*. 2016. Evidence for the sexual origin of heterokaryosis in arbuscular mycorrhizal fungi. Nature Microbiology 1: e16033. 10.1038/nmicrobiol.2016.33 27572831

[nph17530-bib-0047] Rosikiewicz P, Bonvin J, Sanders IR. 2017. Cost‐efficient production of *in vitro Rhizophagus irregularis* . Mycorrhiza 27: 477–486. 28210812 10.1007/s00572-017-0763-2 PMC5486606

[nph17530-bib-0048] Savary R, Dupuis C, Masclaux FG, Mateus ID, Rojas EC, Sanders IR. 2020. Genetic variation and evolutionary history of a mycorrhizal fungus regulate the currency of exchange in symbiosis with the food security crop cassava. ISME Journal 14: 1333–1344. 10.1038/s41396-020-0606-6 PMC7242447 32066875

[nph17530-bib-0049] Savary R, Masclaux FG, Wyss T, Droh G, Cruz Corella J, Machado AP, Morton JB, Sanders IR. 2018a. A population genomics approach shows widespread geographical distribution of cryptic genomic forms of the symbiotic fungus *Rhizophagus irregularis* . ISME Journal 12: 17–30. 10.1038/ismej.2017.153 PMC5739010 29027999

[nph17530-bib-0050] Savary R, Villard L, Sanders IR. 2018b. Within‐species phylogenetic relatedness of a common mycorrhizal fungus affects evenness in plant communities through effects on dominant species. PLoS ONE 13: e0198537. 30462644 10.1371/journal.pone.0198537 PMC6248901

[nph17530-bib-0051] Schmieder R, Edwards R. 2011. Quality control and preprocessing of metagenomic datasets. Bioinformatics 27: 863–864. 21278185 10.1093/bioinformatics/btr026 PMC3051327

[nph17530-bib-0052] Schmieder R, Lim YW, Rohwer F, Edwards R. 2010. TagCleaner: identification and removal of tag sequences from genomic and metagenomic datasets. BMC Bioinformatics 11: e341. 20573248 10.1186/1471-2105-11-341 PMC2910026

[nph17530-bib-0053] Scott TW, Kiers ET, Cooper GA, Dos Santos M, West SA. 2019. Evolutionary maintenance of genomic diversity within arbuscular mycorrhizal fungi. Ecology and Evolution 9: 2425–2435. 30891190 10.1002/ece3.4834 PMC6405528

[nph17530-bib-0054] St‐Arnaud M, Hamel C, Vimard B, Caron M, Fortin JA. 1996. Enhanced hyphal growth and spore production of the arbuscular mycorrhizal fungus *Glomus intraradicis* in and *in vitro* system in the absence of host roots. Mycological Research 100: 328–332.

[nph17530-bib-0055] Steidinger BS, Crowther TW, Liang J, Van Nuland ME, Werner GDA, Reich PB, Nabuurs GJ, de‐Miguel S, Zhou M, Picard N*et al*. 2019. Climatic controls of decomposition drive the global biogeography of forest‐tree symbioses. Nature 569: 404–408. 31092941 10.1038/s41586-019-1128-0

[nph17530-bib-0056] Tatusov RL, Fedorova ND, Jackson JD, Jacobs AR, Kiryutin B, Koonin EV, Krylov DM, Mazumder R, Mekhedov SL, Nikolskaya AN*et al*. 2003. The COG database: an updated version includes eukaryotes. BMC Bioinformatics 4: e41. 12969510 10.1186/1471-2105-4-41 PMC222959

[nph17530-bib-0057] Tisserant E, Malbreil M, Kuo A, Kohler A, Symeonidi A, Balestrini R, Charron P, Duensing N, Frei dit Frey N, Gianinazzi‐Pearson V*et al*. 2013. Genome of an arbuscular mycorrhizal fungus provides insight into the oldest plant symbiosis. Proceedings of the National Academy of Sciences, USA 110: 20117–20122. 10.1073/pnas.1313452110 PMC3864322 24277808

[nph17530-bib-0058] Van der Heijden MGA, Klironomos JN, Ursic M, Moutoglis P, Streitwolf‐Engel R, Boller T, Wiemken A, Sanders IR. 1998. Mycorrhizal fungal diversity determines plant biodiversity, ecosystem variability and productivity. Nature 396: 69–72.

[nph17530-bib-0059] Wang L, Wang S, Li W. 2012. Rseqc: quality control of RNA‐seq experiments. Bioinformatics 28: 2184–2185. 22743226 10.1093/bioinformatics/bts356

[nph17530-bib-0060] Wang N, Thomson M, Bodles WJ, Crawford RM, Hunt HV, Featherstone AW, Pellicer J, Buggs RJ. 2013. Genome sequence of dwarf birch (*Betula nana*) and cross‐species RAD markers. Molecular Ecology 22: 3098–3111. 23167599 10.1111/mec.12131

[nph17530-bib-0061] Wyss T, Masclaux FG, Rosikiewicz P, Pagni M, Sanders IR. 2016. Population genomics reveals that within‐fungus polymorphism is common and maintained in populations of the mycorrhizal fungus *Rhizophagus irregularis* . ISME Journal 10: 2514–2526. 10.1038/ismej.2016.29 PMC5030683 26953600

[nph17530-bib-0062] Yildirir G, Malar CM, Kokkoris V, Corradi N. 2020. Parasexual and sexual reproduction in arbuscular mycorrhizal fungi: room for both. Trends in Microbiology 28: 517–519. 32360097 10.1016/j.tim.2020.03.013

[nph17530-bib-0063] Zhu YO, Sherlock G, Petrov DA. 2016. Whole genome analysis of 132 clinical *Saccharomyces cerevisiae* strains reveals extensive ploidy variation. Genes Genomes Genetics 6: 2421–2434. 27317778 10.1534/g3.116.029397 PMC4978896

[nph17530-bib-0064] Zou X, Du Y, Wang X, Wang Q, Zhang B, Chen J, Chen M, Doyle JJ, Ge S. 2020. Genome evolution in *Oryza* allopolyploids of various ages: insights into the process of diploidization. The Plant Journal 105: 721–735. 33145857 10.1111/tpj.15066

