# Supplementary material for: Generation of unequal nuclear genotype proportions in Rhizophagus irregularis progeny causes allelic imbalance in gene transcription
Source: New Phytol. 2021 Jul 3;231(5):1984–2001. doi: 10.1111/nph.17530 (PMC8457141; doi:10.1111/nph.17530)
Supplement: Supplementary file 1 — Fig. S1 Reproducibility of bi‐allelic (BA) sites in the ddRAD‐sequencing data. Fig. S2 Consistency of bi‐allelic (BA) sites in ddRADseq data and their transcription. Fig. S3 Reproducibility of bi‐allelic (BA) sites in the RNA‐sequencing data. Fig. S4 Allele frequency distributions of diploid (C. albicans and B. nana) and tetraploid (B. × intermedia) species derived from ddRAD‐seq data. Fig. S5 Genes with allele transcription under allelic imbalance. Fig. S6 Reproducibility of mono‐allelic and bi‐allelic expression among three biological replicates of each of the 6 dikaryon single spores sibling lines (SSSLs) of R. irregularis C3. [file NPH-231-1984-s001.pdf]

Supporting Information Figures

Generation of unequal nuclear genotype proportions in *Rhizophagus irregularis* progeny causes allelic imbalance in gene transcription

Chanz Robbins*, Joaquim Cruz Corella*, Consolée Aletti, Réjane Seiler, Ivan D. Mateus, Soon-Jae Lee, Frédéric G. Masclaux, Ian R. Sanders

* These authors contributed equally to this work

Accepted 26th May 2021

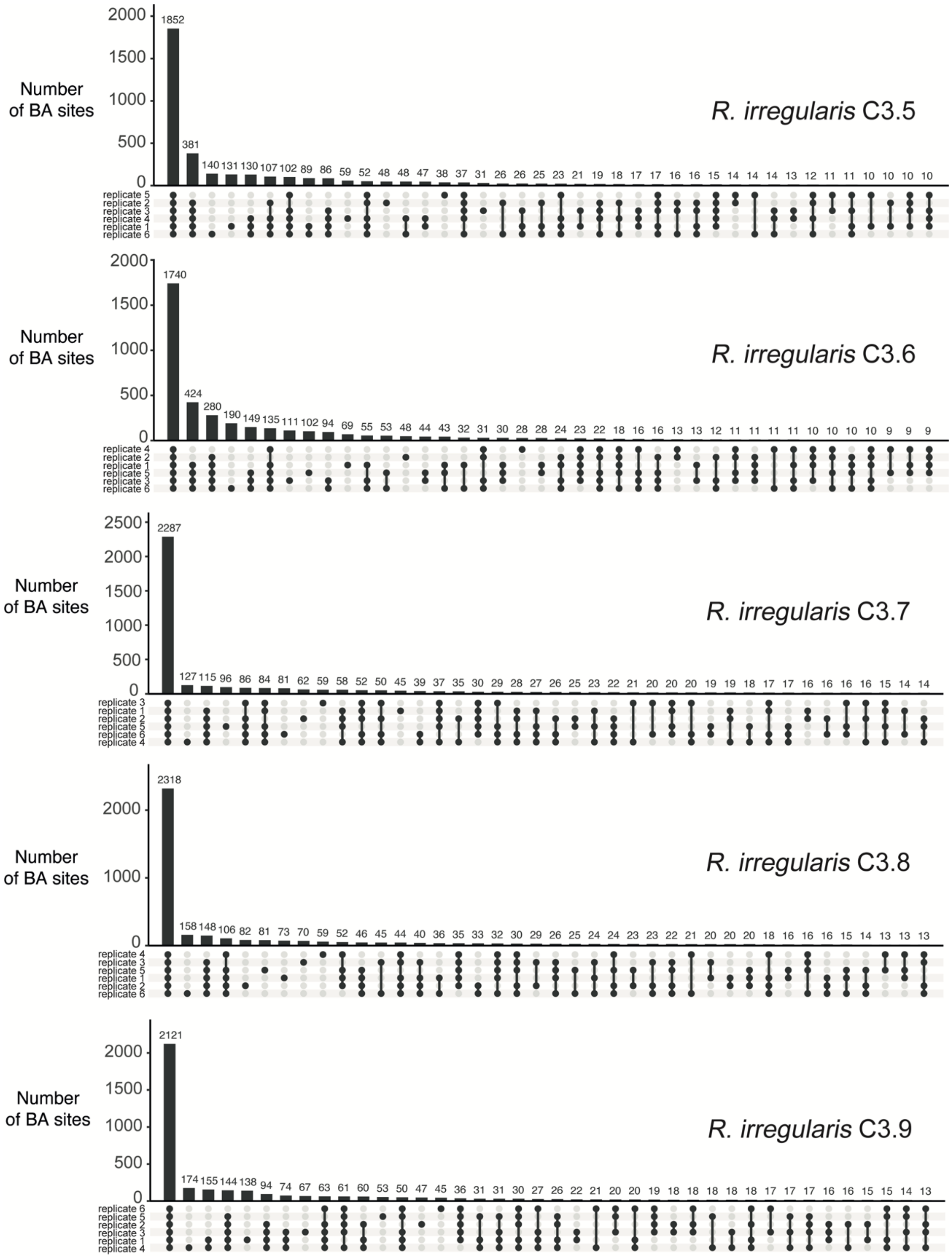

Figure S1: Reproducibility of bi-allelic (BA) sites in the ddRAD-sequencing data among the six replicates in each of the 5 dikaryon Single spores sibling lines (SSSLs) of *R. irregularis* C3. The *y*-axis represents the number of

BA sites commonly detected among the replicates being compared. The replicates considered for comparison are labelled at the left and shown along the horizontal axis by darkened circles indicating they were included in the comparison. Isolates are listed from top to bottom and indicated at the far right.

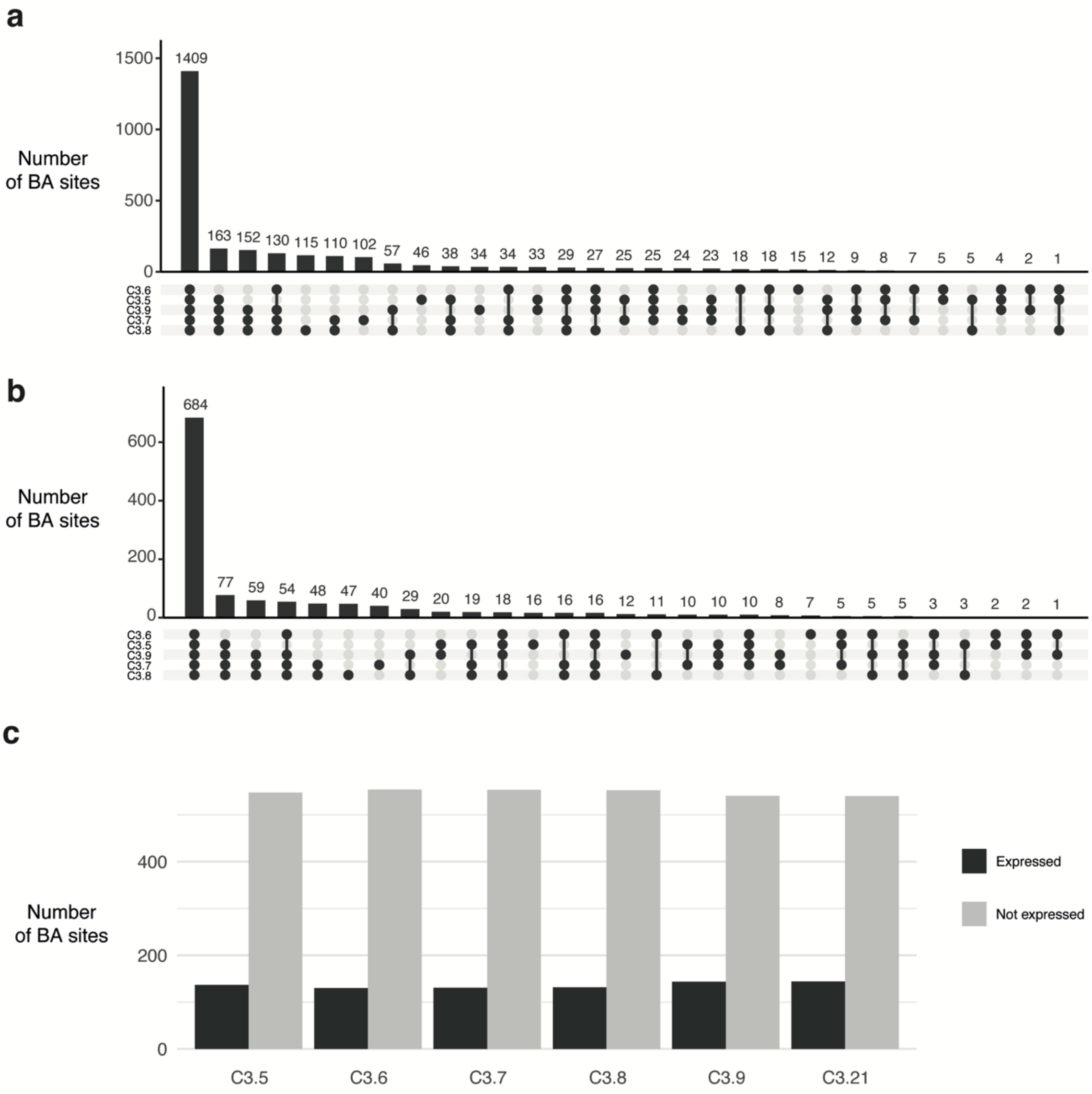

Figure S2: Consistency of bi-allelic (BA) sites in ddRADseq data and their transcription among single spore sibling lines (SSSLs) of *R. irregularis* C3. **(a)** Reproducibility of BA sites in the ddRAD-sequencing data among 5 SSSLs of *R. irregularis* C3. SSSLs being compared are indicated with darkened circles and the number of BA sites is indicated on the *y*-axis. **(b)** Reproducibility of BA sites within coding sequences in the ddRAD-sequencing data among the 5 SSSLs of *R. irregularis* C3. SSSLs being compared are indicated with darkened circles and the number of BA genes is indicated on the *y*-axis. **(c)** Evidence of expression of

the 684 consistent BA sites from the ddRAD-sequencing data in the RNA-sequencing data.

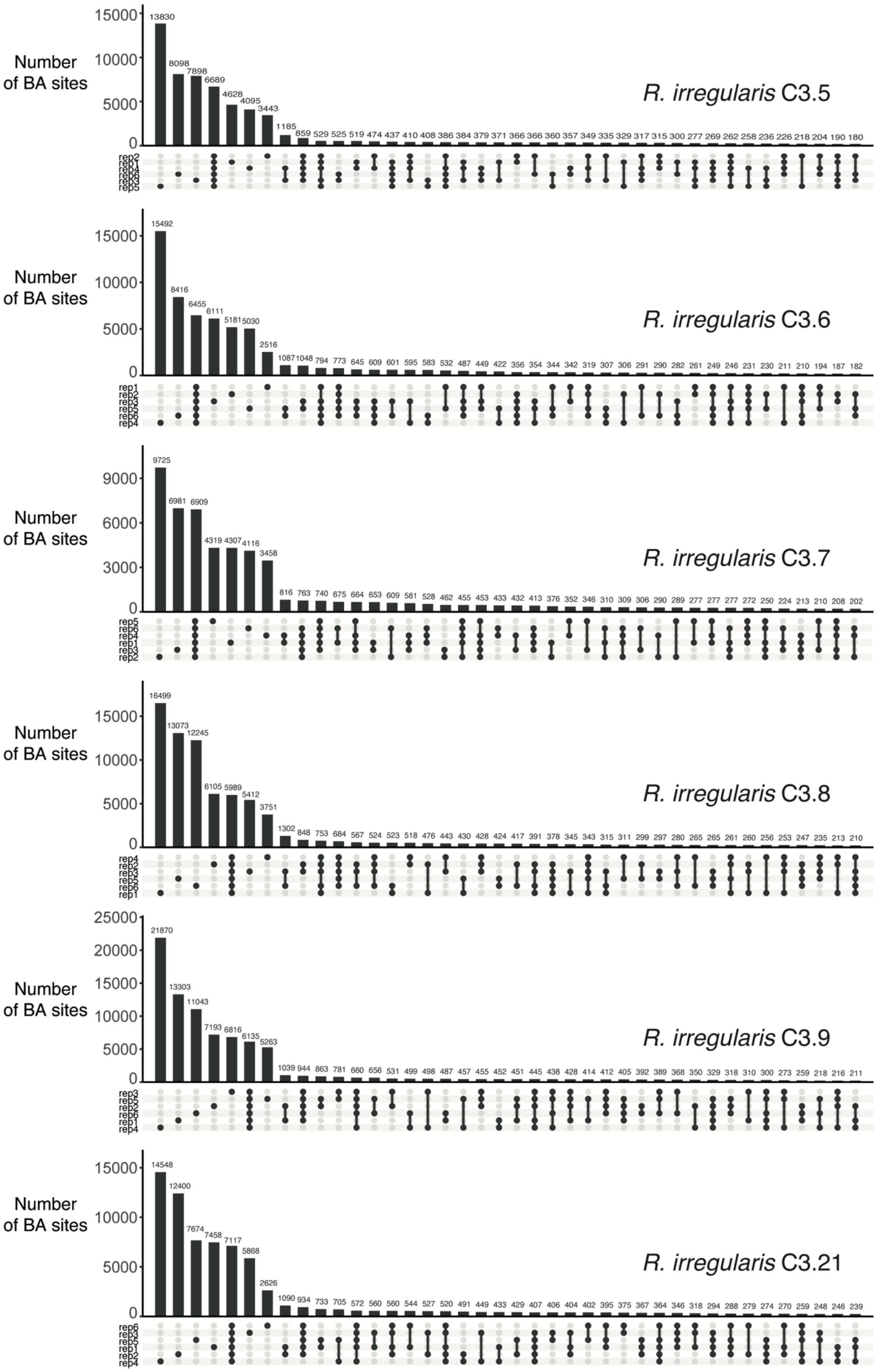
Number of BA sites
R. irregularis C3.5
13830 8098 7898 6689 4628 4095 3443 1185 859 529 525 519 474 437 410 408 386 384 379 371 366 366 360 357 349 335 329 317 315 300 277 269 262 258 236 226 218 204 190 180
rep2 rep1 rep4 rep6 rep3 rep5
Number of BA sites
R. irregularis C3.6
15492 8416 6455 6111 5181 5030 2516 1087 1048 794 773 645 609 601 595 583 532 487 449 422 356 354 344 342 319 307 306 291 290 282 261 249 246 231 230 211 210 194 187 182
rep1 rep2 rep3 rep5 rep6 rep4
Number of BA sites
R. irregularis C3.7
9725 6981 6909 4319 4307 4116 3458 816 763 740 675 664 653 609 581 528 462 455 453 433 432 413 376 352 346 310 309 306 290 289 277 277 277 272 250 224 213 210 208 202
rep5 rep6 rep4 rep1 rep3 rep2
Number of BA sites
R. irregularis C3.8
16499 13073 12245 6105 5989 5412 3751 1302 848 753 684 567 524 523 518 476 443 430 428 424 417 391 378 345 343 315 311 299 297 280 265 265 261 260 256 253 247 235 213 210
rep4 rep2 rep3 rep5 rep6 rep1
Number of BA sites
R. irregularis C3.9
21870 13303 11043 7193 6816 6135 5263 1039 944 863 781 660 656 531 499 498 487 457 455 452 451 445 438 428 414 412 405 392 389 368 350 329 318 310 300 273 259 218 216 211
rep3 rep5 rep2 rep6 rep1 rep4
Number of BA sites
R. irregularis C3.21
14548 12400 7674 7458 7117 5868 2626 1090 934 733 705 572 560 560 544 527 520 491 449 433 429 407 406 404 402 395 375 367 364 346 318 294 288 279 274 270 259 248 246 239
rep6 rep3 rep5 rep1 rep2 rep4

Figure S3: Reproducibility of bi-allelic (BA) sites in the RNA-sequencing data among the six replicates in each of the 6 dikaryon single spore sibling lines (SSSLs) of *R. irregularis* C3. The y-axis represents the number of BA sites commonly detected among replicates being compared. The replicates considered for comparison are labelled at the left and shown along the horizontal axis by darkened circles indicating they were included in the comparison. Isolates are listed from top to bottom and indicated at the far right.

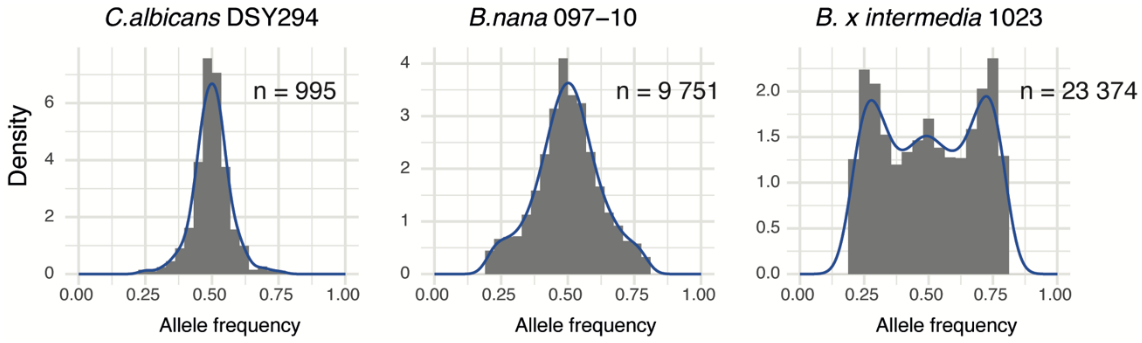

Figure S4: Allele frequency distributions of diploid (*C. albicans* and *B. nana*) and tetraploid (*B. x intermedia*) species derived from ddRAD-seq data. The number of bi-allelic sites analyzed for each species is indicated as (n). Diploid species are expected to exhibit one peak (0.5), and tetraploid species are expected to exhibit three peaks (0.25, 0.5 and 0.75).

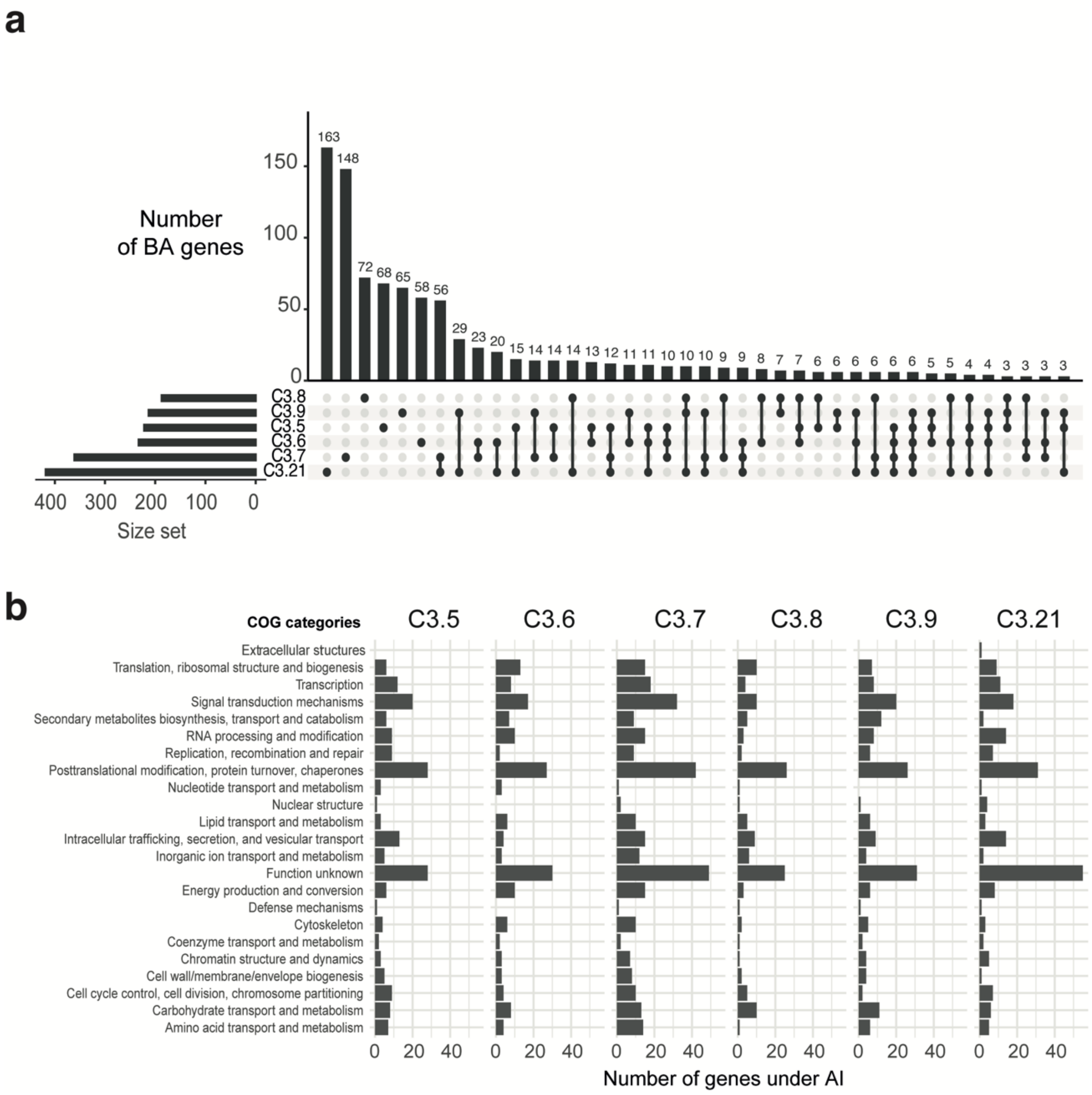

Figure S5: Genes with allele transcription under allelic imbalance. **(a)** Reproducibility of genes under allelic imbalance among the six R. irregularis SSSLs. **(b)** Cluster of orthologous (COG) categories of the genes under allelic imbalance for each *R. irregularis* SSSL.

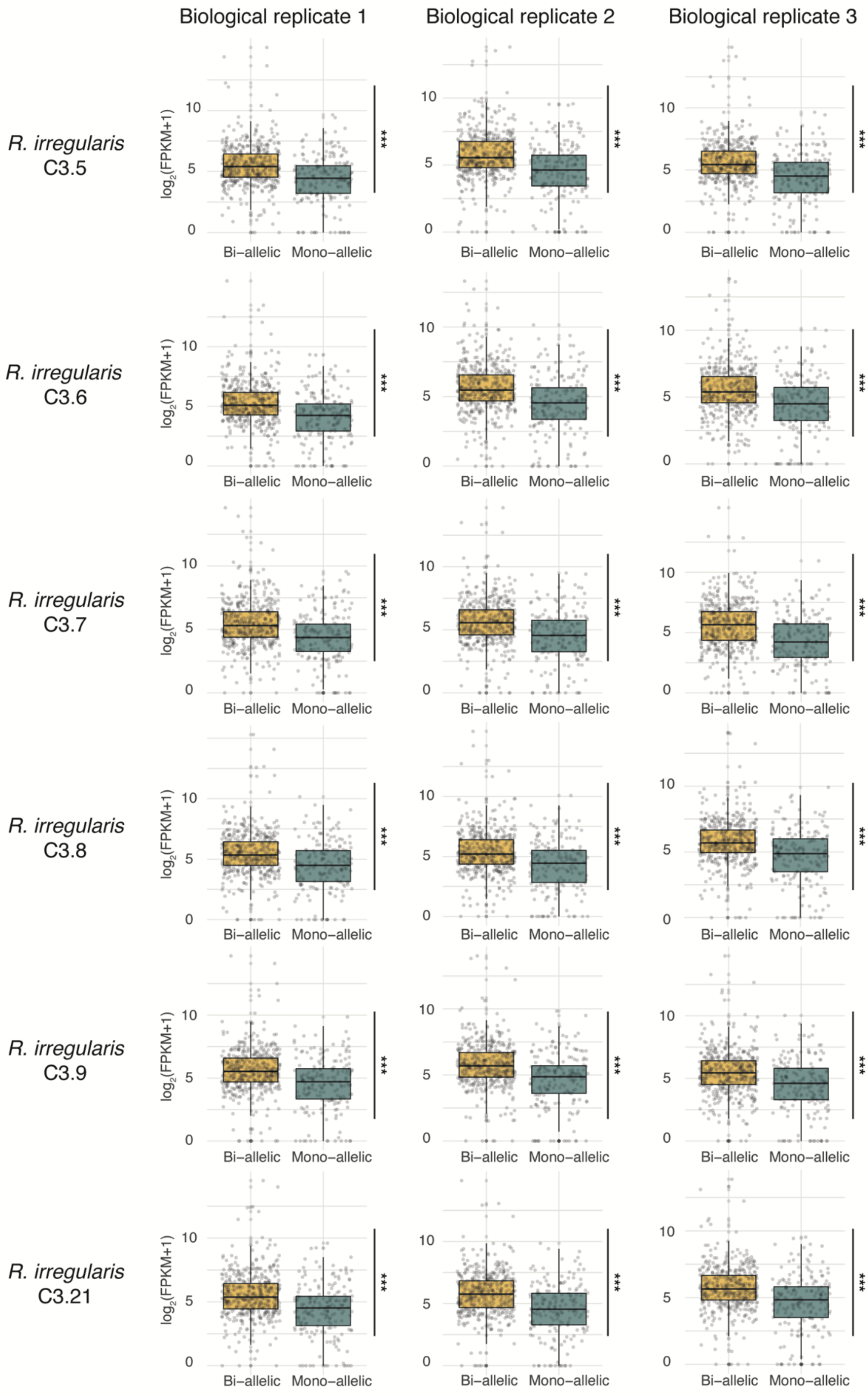

Figure S6: Reproducibility of mono-allelic and bi-allelic expression among three biological replicates of each of the 6 dikaryon single spores sibling

lines (SSSLs) of *R. irregularis* C3. Comparison of the expression levels after a log_2(FPKM+1) transformation, between the 187 reproducible genes exhibiting mono-allelic expression and 187 reproducible genes exhibiting bi-allelic expression, for each biological replicate of each *R. irregularis* SSSL. The three asterisks (***) indicate a statistically significant difference between the medians (Mann-Whitney U-test, with a significance threshold of $p < 0.05$). The horizonal line represents the median, the box represents in the interquartile range and the vertical lines represent the maximum and minimum values.
